# Evaluating the power and limitations of genome-wide association studies in *Caenorhabditis elegans*

**DOI:** 10.1093/g3journal/jkac114

**Published:** 2022-05-10

**Authors:** Samuel J Widmayer, Kathryn S Evans, Stefan Zdraljevic, Erik C Andersen

**Affiliations:** Molecular Biosciences, Northwestern University, Evanston, IL 60208, USA; Molecular Biosciences, Northwestern University, Evanston, IL 60208, USA; Department of Biological Chemistry, University of California—Los Angeles, Los Angeles, CA 90095, USA; Molecular Biosciences, Northwestern University, Evanston, IL 60208, USA; Robert H. Lurie Comprehensive Cancer Center, Northwestern University, Chicago, IL 60611, USA

**Keywords:** *Caenorhabditis elegans*, genome-wide association studies, power, QTL, simulations

## Abstract

Quantitative genetics in *Caenorhabditis elegans* seeks to identify naturally segregating genetic variants that underlie complex traits. Genome-wide association studies scan the genome for individual genetic variants that are significantly correlated with phenotypic variation in a population, or quantitative trait loci. Genome-wide association studies are a popular choice for quantitative genetic analyses because the quantitative trait loci that are discovered segregate in natural populations. Despite numerous successful mapping experiments, the empirical performance of genome-wide association study has not, to date, been formally evaluated in *C. elegans*. We developed an open-source genome-wide association study pipeline called NemaScan and used a simulation-based approach to provide benchmarks of mapping performance in collections of wild *C. elegans* strains. Simulated trait heritability and complexity determined the spectrum of quantitative trait loci detected by genome-wide association studies. Power to detect smaller-effect quantitative trait loci increased with the number of strains sampled from the *C. elegans* Natural Diversity Resource. Population structure was a major driver of variation in mapping performance, with populations shaped by recent selection exhibiting significantly lower false discovery rates than populations composed of more divergent strains. We also recapitulated previous genome-wide association studies of experimentally validated quantitative trait variants. Our simulation-based evaluation of performance provides the community with critical context to pursue quantitative genetic studies using the *C. elegans* Natural Diversity Resource to elucidate the genetic basis of complex traits in *C. elegans* natural populations.

## Introduction

Quantitative trait variation in human populations is abundant and arises from genetic differences between individuals, as well as inputs from the environment. Genetic variation can be statistically linked to phenotypic variation using genome-wide association studies (GWAS). GWAS have uncovered genetic variants that contribute cumulatively to human disease risk and complex trait variation (Visscher *et al.* 2017). However, the most powerful and useful applications of GWAS to complex human traits rely on precise phenotype measurements from hundreds of thousands of individuals. Also, many important sources of variation in disease risk and trait variation cannot be measured ethically, reliably, and with sufficient statistical power in human populations (*e.g*., cellular pathology underlying behavioral traits and variation in diet or xenobiotic exposure underlying metabolic traits). This gap underscores an urgent need for replicable and translatable genome-wide association (GWA) platforms with the added ability to dissect traits that are difficult to assay in humans.

The development of genetic reference populations has become increasingly popular and has facilitated the analysis of complex traits for several organisms, including the *Drosophila* Synthetic Population Resource (King, Macdonald, *et al.* 2012; King, Merkes, *et al.* 2012), *Drosophila* Genetic Reference Panel (Mackay *et al.* 2012), the Collaborative Cross (Churchill *et al.* 2004; Chesler *et al.* 2008; Aylor *et al.* 2011), and BXD (Peirce *et al.* 2004; Ashbrook *et al.* 2021) mouse recombinant inbred line (RIL) panels, outbred mouse populations (Churchill *et al.* 2012; Svenson *et al.* 2012; Nicod *et al.* 2016; Parker *et al.* 2016), the hybrid mouse diversity panel (Bennett *et al.* 2010), outbred rat populations (Rat Genome Sequencing and Mapping Consortium *et al.* 2013; Chitre *et al.* 2020), *Arabidopsis* MAGIC and RILs (Kover *et al.* 2009; Klasen *et al.* 2012), and nested association mapping lines in both maize (Yu *et al.* 2008; McMullen *et al.* 2009) and sorghum (Bouchet *et al.* 2017). These genetic reference populations offer tremendous benefits for quantitative genetics because they take advantage of well characterized genomic resources and the opportunity to collect repeated measurements from diverse genetic backgrounds in controlled environments.

The free-living roundworm nematode *Caenorhabditis elegans* has contributed to discoveries at every level of biology, has rich genomic resources, and can be easily genetically manipulated. Over the past few decades, the number of cataloged genetically unique *C. elegans* wild strains has expanded, giving rise to diverse collections of strains useful for quantitative genetics (Cook *et al.* 2017; Lee *et al.* 2021). For example, both the *C. elegans* Multiparent Experimental Evolution (CeMEE) panel and the *C. elegans* mpRIL population offer fertile ground for quantitative trait locus (QTL) mapping with high-resolution and detection power (Noble *et al.* 2017, 2021; Snoek *et al.* 2019). Although rich in novel haplotypes derived from its founders, alleles segregating within these populations represent only a small fraction of the segregating genetic variants present across the *C. elegans* species. The *C. elegans* Natural Diversity Resource (CeNDR) has expanded to over 500 genetically unique *C. elegans* strains. GWA mapping has repeatedly linked phenotypic variation of all types to alleles segregating among these strains (Ghosh *et al.* 2012; Ashe *et al.* 2013; Cook *et al.* 2016; Laricchia *et al.* 2017; Lee *et al.* 2017, 2019; Zdraljevic *et al.* 2017, 2019; Hahnel *et al.* 2018; Gimond *et al.* 2019; Webster *et al.* 2019; Evans *et al.* 2020; Na *et al.* 2020; Evans, van Wijk, *et al.* 2021; Evans, Wit, *et al.* 2021; Zhang *et al.* 2021). However, GWAS in *C. elegans* has not, to date, been formally evaluated for its power and precision to detect QTL across a range of genetic architectures.

The ability to identify functional natural variation in complex traits in *C. elegans* using GWA is confounded by idiosyncratic genomic features. For instance, adaptation to human-associated habitats is hypothesized to have caused the generation of haplotypes with signatures of selective sweeps among many wild *C. elegans* strains. Within these swept haplotypes, genetic variation is drastically reduced and long-range linkage disequilibrium is high—sometimes stretching over 85% of whole chromosomes (Andersen *et al.* 2012). Approximately 66% of the *C. elegans* strains available in CeNDR contain at least one chromosome of which at least 30% contains the most common identical-by-descent haplotype across the species, which can be categorized as a swept haplotype (Lee *et al.* 2021; Zhang *et al.* 2021). The unintended consequence in GWA mapping is that, if the phenotype of interest happens to segregate with a common swept haplotype, it is likely that insufficient ancestral recombination has occurred across that haplotype to resolve individual candidate loci. In contrast to strains rich in swept haplotypes, divergent strains harbor nearly three times the levels of genetic diversity and often lack signatures of recent selection in spite of recent migration and gene flow (Crombie *et al.* 2019). Furthermore, genetically distinct *C. elegans* strains contain “hyper-divergent” regions (Thompson *et al.* 2015; Lee *et al.* 2021) (regions of the genome characterized by high allelic diversity) that segregate at varying frequencies. These regions are hypothesized to be maintained by balancing selection and are predicted to harbor alleles for biological processes that are crucial for environmental sensing, pathogen responses, and xenobiotic stress responses (Lee *et al.* 2021). These observations suggest that evolutionary biology is inextricable from GWA mapping performance in *C. elegans* and that the conclusions drawn about complex trait variation from these analyses are dictated by the population structure of the mapping population as previously reported for other species (Zhao *et al.* 2007; Kang *et al.* 2008). However, the magnitude of the effect of population structure and segregating hyper-divergent regions on mapping performance has not been quantified. In order to assess how mapping performance varies as a function of population composition, we require an approach that can rapidly simulate GWA mappings and address important caveats unique to *C. elegans* genome biology.

We have developed NemaScan, an open-source pipeline for GWA mapping in *C. elegans*. NemaScan offers two profiles: a mapping profile, where users can supply population-specific variant information and trait values to perform their own analyses on real data, and a simulation profile, where users can supply a variety of parameters to provide baseline performance benchmarks for a past, present, or prospective experiment. These parameters include trait heritability, polygenicity, a minimum minor allele frequency for variants included in the marker set, custom sample populations, and specific regions of interest where QTL are simulated and mapped iteratively. NemaScan makes use of two different formulations of the genomic relationship matrix in attempts to correct for varying types of population structure known to exist across the *C. elegans* species using well established frameworks within the GCTA software suite (Yang *et al.* 2011; Jiang *et al.* 2019). We present empirical estimates of detection power and false discovery rates (FDRs) derived from the simulation profile for GWA mapping across different genetic architectures, and we confirm that GWA mappings in *C. elegans* robustly identify most large-effect QTL. We also demonstrate that GWA performance in *C. elegans*, as in other systems, is improved by both increasing the number of strains tested in a population and reducing the amount of population stratification in the population. Finally, we quantify the precision of GWA mapping when QTL are present on different chromosomes and within hyper-divergent regions that segregate in swept and divergent populations. These performance benchmarks provide the *C. elegans* community with critical context for interpreting the results of ongoing quantitative genetic studies using CeNDR, and in so doing, increase our understanding of the genetic basis of complex traits in *C. elegans*.

## Materials and methods

### The *C. elegans* Natural Diversity Resource (CeNDR)

CeNDR is composed of 1,379 unique *C. elegans* isolates. The process of isolating and identifying unique *C. elegans* strains from the wild, generating whole-genome sequence data, and calling high-quality variants has been described in-depth previously (Crombie *et al.* 2019; Lee *et al.* 2021). Briefly, nematodes that could be unambiguously described as *C. elegans* by both morphological characteristics and ITS2 sequencing were reared, and genomic DNA from these strains (*n = *1,238) was isolated and whole-genome sequenced. High-quality, adapter-trimmed sequencing reads were aligned to the N2 reference genome and single nucleotide variant (SNVs) were called for each strain using GATK. After variant quality filtering, the pairwise genetic similarity of all strains is considered. Strains that share alleles across at least 99.97% of all segregating sites are considered members of the same isotype, of which 540 were identified. In this manuscript, we use the term “strain” to refer to each strain chosen to represent the collection of genetically similar strains within that isotype (*i.e*., the “isotype reference strain”). Because hermaphroditism is the dominant mode of sexual reproduction in *C. elegans* and strains are inbred in the laboratory during the strain isolation process, all strains can be considered fully inbred and homozygous at all loci. All data used in GWA mapping simulations (isotype-level hard-filtered SNVs, sweep haplotype calls, and hyper-divergent region calls) were downloaded from the 20210121 CeNDR release (https://www.elegansvariation.org/data/release/20210121).

### Structure of the NemaScan pipeline

The NemaScan pipeline is written in Nextflow (Di Tommaso *et al.* 2017) and divided into two main profiles: the mapping profile, where users can simply provide collected phenotype data and a full GWAS is conducted, and the simulation profile, where users can provide a set of genetic parameters and quantitative traits are simulated to match these parameters, allowing *ex ante* and *post hoc* evaluation of GWAS performance. The underlying framework of preparing strain variant calls and performing GWAS is similar between both profiles, but each profile offers distinct benefits for different types of users (Supplementary Fig. 1).

#### Mapping profile

This profile allows users to submit trait data collected on *C. elegans* strains and perform GWAS. The trait data are first cross-validated against isotype reference strains present in the VCF file representing the full set of 540 CeNDR isotypes and, if necessary, strain names are altered to reflect isotype reference strain names in the CeNDR release. The VCF is then filtered to the strains present in the submitted trait file and then pruned for variants in *r^2^*_**≥**_ 0.8 within 50 kb windows spaced 10 variants apart across the chromosome and filtered to contain variants with a minor allele frequency greater than or equal to the user-supplied minor allele frequency cutoff. The LD-pruned and minor allele frequency (MAF)-filtered VCF is then used to construct a genomic relationship (kinship) matrix among all strains using the –make-grm and –make-grm-inbred function from GCTA. The methods for constructing the genomic relationship matrix and the benefits of each for association mapping has been described in-depth elsewhere (Jiang *et al.* 2019). At this point, GWAS is then performed using two separate methodologies: fastGWA using a leave-one-chromosome-out (LOCO) approach to constructing the kinship matrix and fastGWA using a kinship matrix designated for inbred model organisms (denoted as INBRED). Both algorithms provide situational advantages for *C. elegans* and were selected based on empirical performance metrics (see *Results*). The user has three threshold choices to determine significance of marker associations: (1) Bonferroni correction using all tested markers (“BF”); (2) Bonferroni correction using the number of independent tests determined by eigendecomposition of the population VCF (“EIGEN”); or (3) any nominal value supplied by the user. SNVs exceeding the user-specified significance threshold are then grouped into QTL “regions of interest,” motivated by the fact that *C. elegans* can be rapidly crossed to generate near-isogenic lines (NILs) harboring small introgressed regions to localize candidates using fine mapping. Regions of interest are determined by finding significantly associated markers within 1 kb of one another or any user-specified distance. Once no more markers meet this criterion, the region of interest is extended on each flank by a user-specified number of markers. We note that demarcating regions of interest in this manner will sometimes mask conditional dependence of adjacent associated markers caused by pervasive LD. Users of NemaScan can provide different parameter values to specify the region of interest depending on the granularity of reporting that they desire. For instance, if the user would like to consider each individual significant marker association as an independent QTL, the grouping and extension parameters can be set to zero. Therefore, default parameters for defining regions of interest in the NemaScan framework are not meant to assign statistically meaningful confidence to all markers within the region but rather provide experimenters with a starting point for genetic crosses designed to fine map QTL and establish causal relationships between individual variants and their trait of interest. The raw mapping results are processed and appended with metadata describing any identified QTL, including trait values for each strain at the top associated marker, the phenotypic variance explained by that QTL, and the start and end positions of the calculated QTL region of interest. The phenotypic variance explained by a QTL is calculated using a simple ANOVA model using the simulated phenotypes as a response and the allelic state of each strain as a factor for the peak associated marker within a region of interest. If any QTL are identified, these regions are passed to a fine-mapping step in which GWAS is rerun using fastGWA with the INBRED kinship matrix against all markers within the QTL interval without any LD pruning. Any identified QTL are also passed to a gene expression mediation analysis in which transcript abundance measurements collected from a select set of *C. elegans* strains are tested as mediators of QTL effects (Evans and Andersen 2020; Zhang *et al.* 2022). Diagnostic plots including Manhattan plots of both whole-genome scans and fine mappings are produced and synthesized into an HTML file to help users prioritize candidate genes and design experiments to validate any detected QTL.

#### Simulation profile

This profile allows users to make flat files required to simulate genetic parameters and automatically downloads the latest CeNDR variant release as a VCF file. Briefly, the user provides files specifying genetic parameters of simulated GWA mappings, including the CeNDR variant set release to use as the source of genetic variants, minor allele frequency cutoff for variant inclusion in the mappings, trait heritabilities, number of causal QTL, QTL effect ranges, and the number of replicate mappings to be performed for each permutation of the previously mentioned parameters. The user may also specify the location of causal QTL using a supplied .bed file, as well as a list of custom strain sets in which GWA mappings are to be simulated. Separately, the user-specified number of causal variants are then sampled from LD-pruned and MAF-filtered VCF and assigned effects sampled from a user-specified effect distribution [either *Uniform [a, b]* (where a = the user-specified minimum effect and b = the user-specified maximum effect) or *Gamma* (*k = *0.4, *θ  *=  1.66)]. Once these effects are assigned to causal variants, phenotype values are then simulated for each of the strains in the supplied population using the –simu-causal-loci function from GCTA and the user-specified trait heritability. The user-specified number of replicates dictates the number of simulated trait distributions within each combination of strain sets, heritability values, and specified number of causal variants. Simulated phenotypes, filtered variants, and the genomic relationship matrix are brought together to perform rapid GWA in the same fashion as the mapping profile.

### Performance assessment

We used the simulation profile of NemaScan to showcase the performance of GWAS in *C. elegans* across a broad parameter space, including trait heritability, degree to which traits are polygenic, sample size and composition, and QTL location in the genome. We cross-referenced simulated causal variants for each mapping and asked whether any detected QTL region of interest overlapped with a simulated causal variant. QTL regions of interest were denoted by the peak association found within the region and assigned the phenotypic variance explained by that peak marker and its frequency in subsequent analyses. The possible outcomes regarding the performance of GWA mapping to detected simulated causal variants were (1) a simulated causal variant exceeded the defined significance threshold and was the peak association within a region of interest, (2) a simulated causal variant exceeded the defined significance threshold but was *not* the peak association within a region of interest, (3) a simulated causal variant did *not* exceed the defined significance threshold but still fell within the calculated QTL region of interest, and (4) a simulated causal variant did not exceed the significance threshold nor fall within a QTL region of interest. For each replicate mapping, we calculated detection power as the number of causal variants that adhered to criteria (1) or (2) and divided them by the total number of causal variants simulated for that mapping. QTL regions of interest that did not contain a simulated causal variant were tabulated as false discoveries, and the FDR was calculated as the number of QTL regions of interest that did not contain a simulated variant divided by the total number of QTL regions of interest for each mapping. For analyses assessing the ability of GWA mappings from different strain sets and population sizes to detect causal variants explaining a particular amount of phenotypic variance, detection power was calculated by first determining the number of causal variants that adhered to criteria (1) or (2) *and* that explained that amount of phenotypic variance. We then divided them by the total number of causal variants simulated that explained the same amount of phenotypic variance across all replicate mappings of each population and then averaged over all populations.

### Demographic characterization of strains

Haplotype data for 540 *C. elegans* strains were obtained from the 20210121 CeNDR release. The degree of swept haplotype sharing among strains was determined in a similar fashion to that previously described (Crombie *et al.* 2019; Lee *et al.* 2021; Zhang *et al.* 2021). Briefly, the length of every haplotype present in each strain was recorded, and if regions sharing the most common haplotype were longer than 1 Mb, these haplotypes were recorded as swept haplotypes. Only swept haplotypes on chromosomes I, IV, V, and X were considered in strain classification because selective sweeps are not found on chromosomes II and III among the set of 540 strains in this CeNDR release. If swept haplotypes composed ≥30% of the length of these chromosomes, that chromosome was considered swept. Swept strains were determined as those strains that contain at least one swept chromosome, and divergent strains are those strains that do not. In total, 357 swept and 183 divergent strains were identified. Some populations used in simulations were constructed by sampling among these swept and divergent strains (Fig. 3), and others were sampled from the overall collection of 540 strains (Figs. 2 and 3). In simulations comparing QTL simulated in hyper-divergent regions from those simulated outside of such regions, we filtered the entire strain set to those containing at least 37 hyper-divergent regions, regardless of their population frequency or distribution. This hyper-divergent region cutoff was selected to yield equally sized swept (*n = *182 strains) and divergent populations (*n = *183 strains) without producing a swept population devoid of hyper-divergent regions entirely, which could confound performance of simulated QTL in these regions with genome-wide genetic stratification. Dendrograms representing population differentiation were constructed for these swept and divergent populations by filtering genetic variants identically to NemaScan and passing these variant calls to vcf2phylip (Ortiz 2019) and QuickTree (https://github.com/khowe/quicktree).

### Statistical testing

Significant differences in performance among experimental factors were determined using both parametric and nonparametric specifications of power or empirical FDR as a response. Simulation regimes where only one QTL was specified for each simulated mapping caused a binary distribution of power output, and differences in performance as a function of experimental factors were determined using the Kruskal–Wallis test. Differences between all pairwise contrasts of factor levels were determined using the Dunn’s test. In cases where multiple experimental factors were considered simultaneously (*e.g*., whether mapping strain set and the location of the single simulated QTL *interacted* to determine performance), factors were combined to make an aggregate factor and tested using the Kruskal–Wallis test. When the specified number of QTL were greater than one, differences in performance as a function of single and multiple factors were determined using the one-way ANOVA and two-way ANOVA tests, respectively, and followed up with *post hoc* tests using Tukey’s HSD.

## Results

### GCTA software improves GWAS power and precision

A previous GWA mapping workflow, cegwas2-nf (Zdraljevic *et al.* 2019), was built on the foundation of kinship matrix specification using EMMA or EMMAX (Kang *et al.* 2008, 2010) implemented by R/rrBLUP (Endelman 2011) as the association mapping algorithm. However, with the advent of more efficient and flexible algorithms, we wondered whether GCTA offered better performance. We first compared available algorithms used for fitting linear mixed models and estimating kinship among individuals in the GWA mapping. Simulations were performed using 4 different association mapping algorithms to generate the kinship matrix, of which three are different implementations of association mapping using GCTA software using linear mixed model association analysis (fastGWA) (Yang *et al.* 2011; Jiang *et al.* 2019). (1) EMMA: GWA mapping using R/rrBLUP fits a kinship matrix and performs association using variance components using the “P3D = TRUE” option; (2) standard approach using –make-grm fits a sparse kinship matrix using all chromosomes (fastGWA-lmm-exact); (3) –make-grm-inbred fits a sparse kinship matrix tailored toward populations composed of inbred organisms (fastGWA-lmm-exact-INBRED); (4) –mlma-loco fits a kinship matrix constructed using all chromosomes except for the chromosome harboring the tested genetic variant (“LOCO”) (fastGWA-lmm-exact-LOCO**)**. This method of constructing the genomic relationship matrix attempts to increase QTL detection power by excluding the tested variant, and others in strong LD, from the covariance matrix in order to reduce the effects of “proximal contamination,” or the tendency of that variant’s inclusion in the kinship matrix to increase the likelihood of the null hypothesis of association (Listgarten *et al.* 2012). This correction causes a theoretical increase in power to detect QTL for which effects are, in part, explained by population stratification. The statistical properties of each mapping algorithm have been reported elsewhere (Yang *et al.* 2011; Jiang *et al.* 2019).

We next used convenient features offered by GCTA to simulate quantitative traits (–simu-qt) and assign effects to QTL (–simu-causal-loci) across a panel of real *C. elegans* genomes. To begin, we used a population of 203 isolates that were previously measured for susceptibility to albendazole (Hahnel *et al.* 2018). We simulated 50 quantitative traits with increasing narrow-sense heritability (the proportion of phenotypic variance explained by specific genetic differences between strains, *h*^2^), ranging from 0.1 to 0.9, supported by either a single QTL or five independent QTL. Each QTL was assigned a large effect size sampled from a uniform distribution (Supplementary Fig. 2) to increase the likelihood that at least one true QTL was detected in each simulation.

**Fig. 1.**
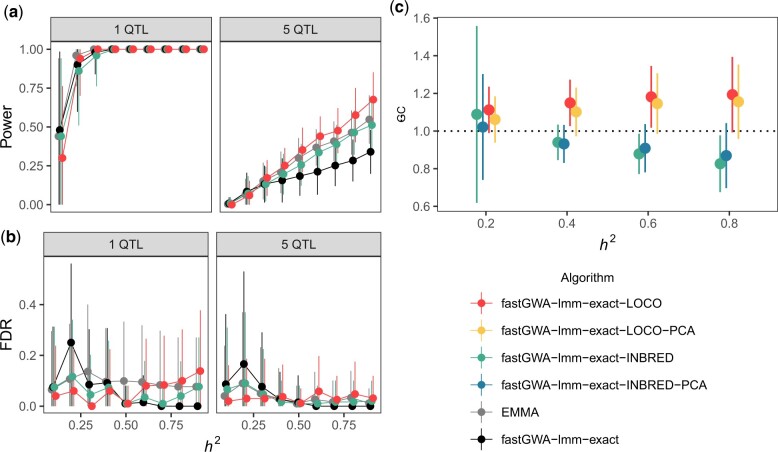
GCTA-based kinship matrix construction and GWAS outperforms EMMA. Estimates of power (a), and FDR (b), and genomic inflation (λ_GC_) (c) of simulated mappings as a function of the narrow-sense heritability (*x*-axis) and the number of underlying QTL (strip titles). Simulations performed using EMMA, which undergirded cegwas2-nf (Zdraljevic *et al.* 2019), were compared with different formulations of kinship matrices and linear mixed model-based GWA implemented by GCTA (Yang *et al.* 2011; Jiang *et al.* 2019). In (c) metrics obtained from mappings that used indicated algorithms but also fitting the first eigenvector of the kinship matrix obtained from principal components analysis are also denoted.

We measured the statistical power and the empirical FDR of each association mapping workflow across varying levels of trait heritability and for traits supported by either one or five QTL applying the Bonferroni correction. We observed that GCTA-based workflows were frequently more powerful than EMMA for each simulated genetic architecture (Fig. 1a). When mapping a single causal QTL, we observed that all algorithms exhibited nearly perfect power when that QTL explained at least 30% of the phenotypic variance (Kruskal–Wallis test, *P* ≥ 0.295). However, when traits were supported by five QTL, power varied among algorithms and increased as a function of trait heritability. When *h*^2^ < 0.4, the algorithms exhibited no significant differences in detection power (Kruskal–Wallis test, *P* ≥* *0.276). When *h*^2^_**≥**_ 0.4, algorithms diverged in performance, fastGWA-lmm-exact and fastGWA–lmm-exact-INBRED generally exhibited lower power than both the EMMA and fastGWA-lmm-exact-LOCO (Dunn test, *P*_adj__**≤**_ 0.01385). Furthemore, LOCO exhibited significantly greater power than EMMA for traits with *h*^2^ > 0.7 (Dunn test, *P*_adj__**≤**_ 0.00826) (Supplementary Table 2). We also observed only modest differences in empirical FDRs among algorithms at different trait heritabilities, among them being that LOCO and fastGWA-lmm-exact-INBRED often exhibited lower empirical FDR than both EMMA and fastGWA-lmm-exact (Fig. 1b;Supplementary Table 3). These results indicated that some mapping algorithms implemented by GCTA have equal or greater power for QTL detection and lower FDR in *C. elegans* than the previous implementation of GWA mapping using EMMA.

We observed that GWA mapping power using kinship matrices constructed using GCTA were either greater than or equal to that obtained using EMMA with comparable rates of false discovery. We also evaluated the amount of genomic inflation provided by these algorithms in mappings using the whole CeNDR collection (*n = *540) with and without including the first eigenvector from principal component decomposition of the kinship matrix. Mappings performed using fastGWA-lmm-exact-INBRED consistently yielded genomic inflation factors below 1, while mappings performed using fastGWA-lmm-exact-LOCO often yielded genomic inflation factors exceeding 1 and significantly different between those performed with and without PCA (1-way ANOVA, Tukey HSD, *P*_adj_ = 0.013), suggesting that fastGWA-lmm-exact-INBRED more effectively and robustly corrects for systemic effects of population stratification (Fig. 1c). Both of these methodologies offer complementary benefits for *C. elegans* GWAS; fastGWA-lmm-exact-LOCO offered greater detection power than our previous pipeline for highly heritable traits, and fastGWA-lmm-exact-INBRED tended to reduce genomic inflation in the entire strain set compared with fastGWA-lmm-exact-LOCO. We therefore chose to perform more extensive simulation evaluation with the more conservative fastGWA-lmm-exact-INBRED kinship matrix construction algorithm. Mapping results provided using CeNDR include results derived from both GCTA-based methodologies with metadata if researchers prefer the handling of the genomic relatedness from one algorithm over the other. These complementary outputs integrated into distinct simulation and mapping profiles is the foundation of our new GWA mapping workflow, called NemaScan.

### Genetic architecture dictates the spectrum of QTL detection using GWA mapping

In order to quantify the ability of NemaScan to identify QTL in natural populations of wild strains, we performed simulations making changes to the genetic architectures of simulated traits. First, simulated QTL effects were drawn from a *Gamma* (*k = *0.4, *θ  *= 1.66) distribution, conforming to the assumption that the natural genetic variants underlying complex traits and adaptation primarily contribute small phenotypic effects but occasionally exert moderate or large effects (Supplementary Fig. 3). Second, because experimenters have limited control over noise in phenotype measurement and, therefore, the narrow-sense heritability of their trait of interest, traits were simulated with *h*^2^ = 0.2, 0.4, 0.6, or 0.8. For each heritability specification, traits were either supported by 1, 5, 10, 25, or 50 QTL to examine GWA performance across a broad spectrum of genetic architectures. Third, we simulated each of these genetic architectures in the complete set of 540 wild isolates currently available from CeNDR to determine the expected performance in the theoretical case where every available genetic background is assayed for a phenotype of interest. Significant QTL were determined using the Bonferroni correction.

**Fig. 2.**
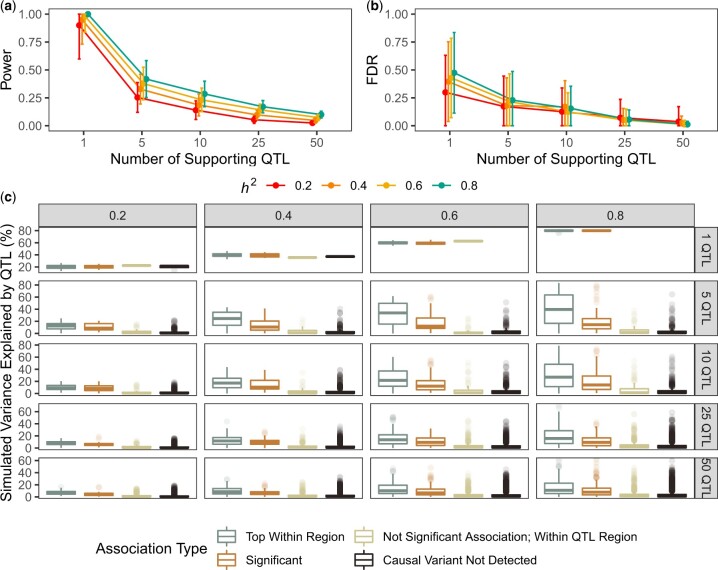
Performance benchmarks for GWAS of complex traits. Estimates of power (a) and FDR (b) as a function of the narrow-sense heritability [0.2, 0.4, 0.6, 0.8] and number of causal QTL (ranging from 1 to 50 QTL) underlying quantitative traits (*x*-axis). c) The empirical phenotypic variance explained by each simulated QTL among all architecture regimes, broken out by whether the causal QTL was the top association within a QTL region of interest, significant (and thereby exceeding the threshold of significance by multiple testing), not a significant association but residing within the QTL region of interest, or outside any region of interest. Lines stretching from each point represent the SD of the performance estimate among all replicate mappings in (a) and (b).

We observed that detection power decreased as a function of the number of supporting QTL for each simulated trait, regardless of its heritability. In the simplest case where a single QTL accounted for all of the phenotypic variance, mappings exhibited at least 90% power to detect it on average. However, detection power decreased as simulated trait complexity increased, especially for less heritable traits (Fig. 2a). NemaScan exhibited only 34.4% power to detect 5 QTL architectures and only 6.2% power to detect 50 QTL architectures, corresponding to detecting on average 1.72 true QTL out of 5 or 3.08 true QTL out of 50, respectively. Depending on the number of simulated QTL, detection power increased by between 1.5-fold (5 QTL) to 4-fold (50 QTL) magnitude when trait heritability was increased from 0.2 to 0.8. The empirical FDR also decreased as a function of genetic complexity (Fig. 2b). Mappings of 5 QTL architectures produced a mean FDR of 20.0%, and mappings of 50 QTL architectures produced a mean FDR of 2.06%. Among traits supported by the same number of QTL, FDR increased with trait heritability but to a much lesser extent than detection power. These results demonstrated that the quantity and effect sizes of underlying QTL and the relative contribution of genetics to phenotypic variation alter the performance of GWA mappings in *C. elegans* similarly to other model organisms. By quantifying increases in power and FDR across various genetic architectures, we also provide performance benchmarks for GWA mappings in *C. elegans* and emphasize that obtaining more precise phenotype measurements, and thereby reducing environmental noise, improves the prospects of QTL detection across *C. elegans* strains.

In *C. elegans* as well as other systems, the power to detect causal alleles underlying QTL in natural populations is limited in part by their frequency and effect size, which together contribute to the fraction of phenotypic variance explained by that QTL. We calculated the phenotypic variance explained by each causal QTL across all simulations and found that true positive QTL (simulated QTL with significant trait associations) explained more phenotypic variance than false negative QTL (causal QTL without significant trait associations) within all combinations of trait heritability and polygenicity regimes (one-way ANOVA, Tukey HSD, *P*_adj_ < 0.05) except for 1 QTL and *h*^2^ = 0.2 (one-way ANOVA, Tukey HSD, *P*_adj__**≥**_ 0.871) (Fig. 2c). The median simulated variance explained by top hits in polygenic architecture simulations ranged from 6.76% (*h*^2^ = 0.2; 50 QTL) to 39.5% (*h*^2^ = 0.8; 5 QTL), and the median simulated variance explained by false negative QTL consistently remained below 2%. When markers with the highest statistical association within a region of interest were also the causal markers, they explained significantly more phenotypic variance than significantly associated causal markers that were not peak associations (one-way ANOVA, Tukey HSD, *P*_adj_ < 0.05), except for traits supported by 1 QTL (one-way ANOVA, Tukey HSD, *P*_adj__**≥**_ 0.815). We conclude from these patterns that, as in other model systems (Yu *et al.* 2008; King, Macdonald, *et al.* 2012; Klasen *et al.* 2012; King and Long 2017; Keele *et al.* 2019), GWA mapping in *C. elegans* is able to robustly detect QTL across traits of varying heritability.

### Sample size and population structure modulates the sensitivity of GWA mapping

**Fig. 3.**
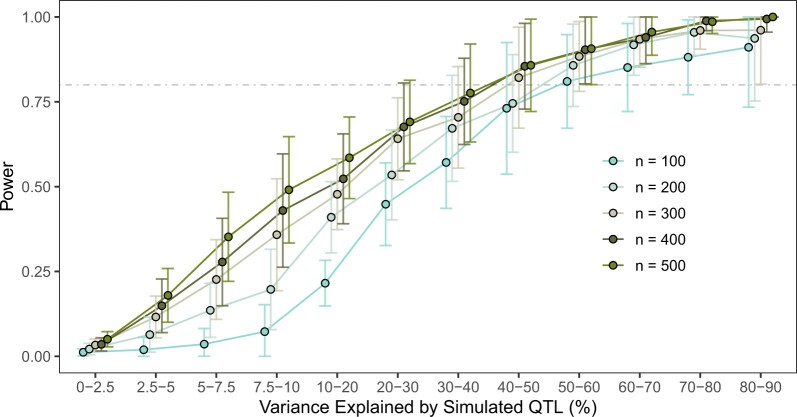
Impact of sample size on sensitivity of QTL detection. Power estimates (a) for GWA mappings conditioning on the variance explained by underlying QTL as a function of sample size and strain selection are shown. The corresponding breakdown of the abundance of QTL explaining increasing phenotypic variance (b) and the MAFs (c) of these QTL are shown. Fifty populations at each sample size were subsampled from CeNDR, each undergoing 50 GWAS simulations with *h*^2^ = 0.8 and 5 underlying QTL with effects sampled from *Gamma* (*k = *0.4, *θ  *= 1.66).

**Fig. 4.**
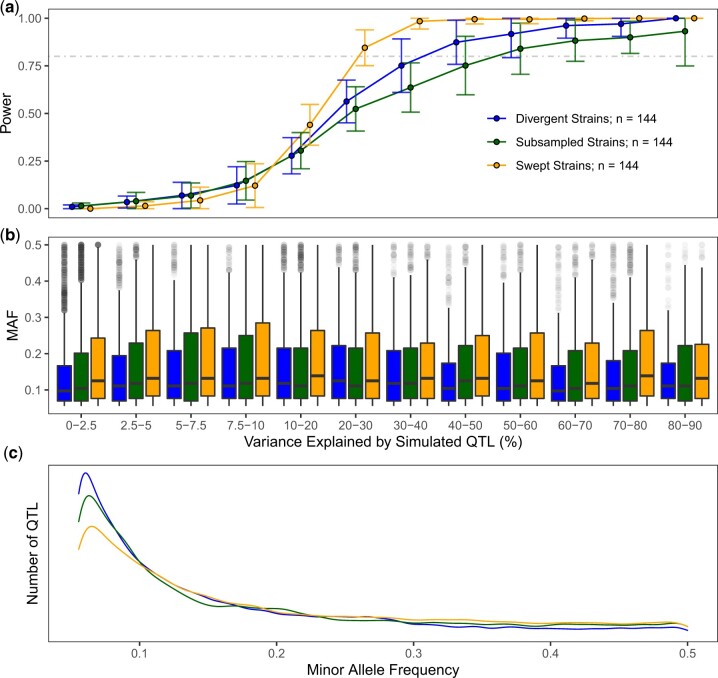
Population composition alters performance and underlying distribution of variants. The fraction of simulated QTL detected by GWA (a) and their minor allele frequencies (b) are plotted as a function of the variance they explain and strain selection. c) The underlying distributions of minor allele frequencies and effects of all simulated QTL for each population are displayed. Populations of *n *=* *144 were sampled from swept strains, divergent strains, or the entire CeNDR collection. Traits were simulated with a heritability of 0.8 and 5 underlying QTL effects sampled from *Gamma* (*k = *0.4, *θ  *= 1.66).

**Fig. 5.**
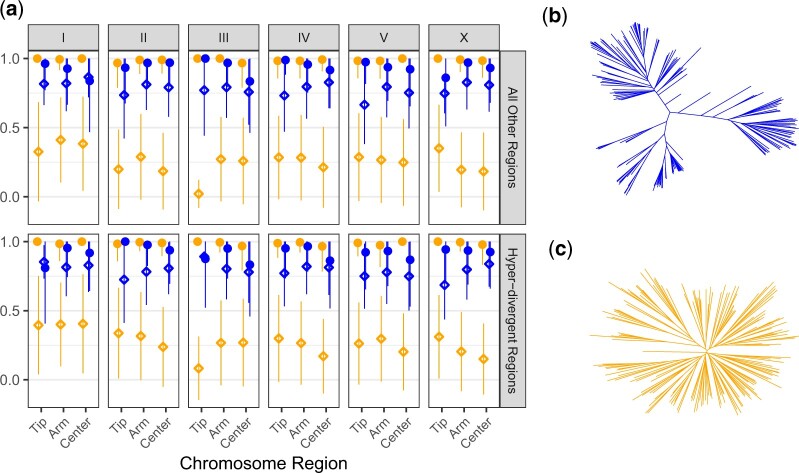
Evolutionary history dictates the fine-scale landscape of GWA performance. a) The mean fraction of simulated QTL detected by GWA (circles) and the empirical FDR (diamonds) colored by sample population, each of which were derived of 183 divergent strains (b) or 182 swept strains (c). Facets correspond to different genomic locations where QTL were simulated. Horizontal facets distinguish simulations of QTL within hyper-divergent regions with respect to the N2 reference genome, or within all other loci. Vertical facets delineate subsets of these horizontal facets on each chromosome. Within each block, performance is separated between the low-recombination centers and tips or high-recombination arms of the denoted chromosome.

**Fig. 6.**
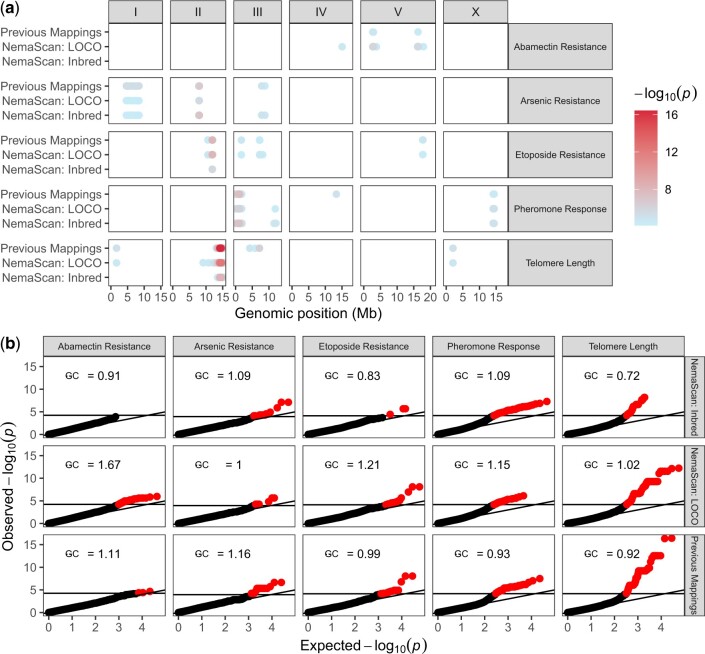
GWA mapping with NemaScan recaptures previously validated QTVs. a) Significant genetic associations are shown genome-wide for five quantitative traits that were remapped using the 20210121 CeNDR release both with cegwas2-nf (“Previous Mappings”) and NemaScan, and the strength of the association is displayed in the right-hand panel. b) Quantile-quantile plots of all −log transformed *P*-values from cegwas2-nf, NemaScan using INBRED kinship matrix construction, and NemaScan using LOCO kinship matrix construction are plotted against their expected rank, with the horizontal line in each panel indicating the trait-specific multiple testing correction significance threshold. Genomic inflation (λ_GC_) calculated from each mapping is displayed in the upper left of each panel.

A common practical limitation of the scope and performance of any GWAS is the size of the sample population for which traits have been measured. *Caenorhabditis elegans* GWA mappings are no exception, despite high-throughput platforms becoming more commonplace in studies of natural phenotypic variation (Yemini *et al.* 2013; Andersen *et al.* 2015). We quantified the detection power of NemaScan when applied to complex traits given the finite sampling potential of a typical GWA experiment. To accomplish this simulation, we subsampled the 540 CeNDR isolates at five different depths (*n = *100, 200, 300, 400, or 500) 50 times each. We then measured the sensitivity of GWA mappings to detect simulated QTL according to the phenotypic variance that they explained by grouping simulated QTL into bins representing increasing influence on trait variation. Among all QTL simulated, we found no clear differences in the minor allele frequencies of identifiable variants among populations of different sizes (Supplementary Fig. 4). For these simulations and all others where the composition and/or size of mapping populations was a dependent variable in the analysis (Figs. 3–5) or not held constant across traits (Fig. 6), we applied the EIGEN multiple-testing correction threshold to determine significant QTL, capitalizing on its ability to increase power within individual mapping simulation replicates by reducing the number of independent tests.

We first observed that, as expected, overall detection power generally increased as a function of sampling depth. The average power to detect 5 QTL among 100 subsampled strain mappings was 0.27_** ± **_0.13 (∼1 QTL out of 5), increasing to 0.41_** ± **_0.17 (at least 2 QTL out of 5) among 500 subsampled strain mappings (Table 1). The observation of ∼41% power to detect 5 QTL at *h*^2^ = 0.8 among 500 subsampled strains is consistent with our previous simulation results (Fig. 2a). We also observed that the impact of increasing sample size was most striking when considering the sensitivities of mappings to detect QTL with smaller effects (Fig. 3). Both 100- and 500-strain mappings had >80% power to detect QTL that explained >50% of the phenotypic variance. However, the power of 500-strain mappings to detect QTL explaining as little as 7.5% of the phenotypic variance (0.49_** ± **_0.16) was nearly seven times greater than that of 100-strain mappings (0.07_** ± **_0.08) (Supplementary Table 4). These results indicate that power to detect QTL with large effects (*i.e*., those QTL explaining >20% of the phenotypic variance) increased only marginally with increasing sampling depth, and power to detect QTL with smaller effects improves significantly by adding more strains to mapping populations.

**Table 1.** Power and FDR estimates for GWA mappings performed with subsampled populations of increasing depth.

Sample size	Power	FDR
100	0.27 ± 0.13	0.63 ± 0.27
200	0.32 ± 0.14	0.49 ± 0.30
300	0.36 ± 0.16	0.38 ± 0.29
400	0.38 ± 0.16	0.31 ± 0.28
500	0.41 ± 0.17	0.27 ± 0.26

Traits were simulated with a heritability of 0.8 and 5 underlying QTL effects sampled from *Gamma* (*k = *0.4, *θ  *= 1.66).

We then measured GWA mapping performance in sets of strains that were distinguished by presence of haplotypes shaped by past selective sweeps (Andersen *et al.* 2012; Crombie *et al.* 2019; Zhang *et al.* 2021). Using the criterion of whether strains harbored at least 1 chromosome composed of at least 30% swept haplotypes, we divided the 540 strains into 2 groups: “swept” strains (*n *=* *357) and “divergent” strains (*n *=* *183). We then simulated and mapped 50 quantitative traits supported by 5 QTL and *h*^2^ = 0.8, and QTL effects were once again sampled from a *Gamma* (*k = *0.4, *θ  *= 1.66) distribution. We performed these simulations using populations of equal sampling depth (*n = *144) from swept strains, divergent strains, and 144 randomly sampled strains from the entire CeNDR strain collection.

We observed that strain composition has a large impact on the sensitivity with which QTL explaining varying amounts of phenotypic variance are detected (Fig. 4a;Supplementary Table 5). Two patterns emerged from these results. First, swept populations exhibited greater detection power than other populations for QTL that explained >10% of the phenotypic variance. Second, for QTL explaining >20% of the phenotypic variance, populations assembled without regard for selective sweep haplotypes exhibited lower power than both swept and divergent populations, despite the fact that divergent populations are enriched for low frequency alleles (Fig. 4b). Furthermore, the differences in power between these populations cannot be explained by underlying differences in the amount of phenotypic variance explained by simulated QTL (Fig. 4c). These simulated mapping results provide evidence that strain choice as well as sampling depth dictate the realized genetic architecture of *C. elegans* quantitative traits, as previously shown for other species.

### Fine-scale genomic landscape of GWA performance

Selective sweep and hyper-divergent region haplotype frequencies and distributions vary across wild strains, motivating us to ask whether heterogeneity in GWA sensitivity among populations with different demographics can be partly explained by the chromosomes on which QTL are located and whether these QTL are also located in hyper-divergent regions. In order to assess these points, we simulated 100 mappings of a single QTL with a defined effect size in a population of 182 swept strains and a population of 183 divergent strains. For each set of 100 mappings, the locations of simulated QTL were assigned to (1) a particular region of the chromosome (tips, arms, or centers) and (2) within or outside of hyper-divergent regions. We simulated these mappings across 3 different heritabilities, but for display purposes, performance was aggregated across these ranges for each chromosome, chromosomal region, and hyper-divergent region assignment type. We observed several critical differences in mapping performance across different regions of the genome and between divergent and swept mapping populations (Fig. 5a).

Power to detect QTL was significantly lower among divergent strains than swept strains across all chromosomes, regardless of whether QTL were in hyper-divergent regions, arms, or centers of the chromosome (Kruskal–Wallis test; *P < *0.0084). However, we also observed subtle differences in the relative detection power for QTL within these classes within certain chromosomes (Supplementary Table 6). The empirical FDR of mappings was significantly greater in mappings among divergent strains than swept strains regardless of the location of simulated QTL (Kruskal–Wallis test; *P < *0.00001). These differences are likely caused by the large extent to which the divergent population was structured into distinct clusters (Fig. 5b), and the swept population much closely approximates a star phylogeny because most variation in the population segregates on a much more common genetic background of swept haplotypes (Fig. 5c). These results confirm a clear effect of population stratification and evolutionary history in *C. elegans*, as previously shown for other species, on both genome-wide precision and local detection power of GWA mapping.

We also investigated whether certain genomic regions provided varying performance for GWA mapping in *C. elegans*. Within the swept population, we observed no significant differences in power to detect QTL simulated in hyper-divergent regions nor on chromosome arms compared with centers (Kruskal–Wallis test; *P = *0.2132). In contrast, power to detect QTL within the divergent population differed subtly as a function of whether they were simulated in hyper-divergent regions or different parts of the chromosome (Supplementary Table 7). For example, power to detect QTL simulated in the centers of chromosomes was significantly lower than on the tips or arms of chromosomes (Dunn test, *P*_adj_ < 0.0396). Once again, the empirical FDR among hyper-divergent region types and different chromosomal regions varied significantly within both the divergent and swept strain set (Kruskal–Wallis test; *P < *0.00001) (Supplementary Table 8). We also observed limited cases where, when QTL were simulated within them, regions of certain chromosomes yielded differences in detection power, primarily among divergent strains (Supplementary Table 9). For example, power to detect QTL simulated within hyper-divergent regions in the center of chromosome III was significantly lower compared with hyper-divergent regions in the centers of chromosomes II, V, and X (Dunn test, *P*_adj__**≤**_ 0.0065). Empirical FDR varied significantly among chromosomes in several instances among both divergent and swept strain sets (Kruskal–Wallis test; *P < *0.05) (Supplementary Table 10). Taken together, these results demonstrate that subtle differences in GWA mapping performance can be explained in part by the interaction of stratified populations of *C. elegans* strains and the recombination landscape of *C. elegans* genomes.

### NemaScan recapitulates previously validated genetic associations

Previous work has used GWA mappings to identify QTL and subsequently identify quantitative trait variants (QTV) in *C. elegans* (Evans, van Wijk, *et al.* 2021). In order to test whether NemaScan performs similarly to cegwas2-nf, the previous mapping pipeline (https://github.com/AndersenLab/cegwas2-nf) that used the EMMA algorithm (Kang *et al.* 2008) implemented by R/rrBLUP (Endelman 2011), we remapped five quantitative traits using both cegwas2-nf and NemaScan. We remapped these traits using both the INBRED and LOCO approaches, asking whether it yielded similar results with real data. Although our evaluation of performance using simulation focused on mappings using the INBRED approach, results from both methodologies are generated as part of the standard output of the mapping profile. Raw trait files were downloaded from the Supplementary Materials for each published mapping (Cook *et al.* 2016; Zdraljevic *et al.* 2017, 2019; Lee *et al.* 2019; Evans, Wit, *et al.* 2021) and remapped using the 20210121 CeNDR release VCF. The experimentally validated QTL underlying each trait were mapped using both cegwas2-nf and NemaScan (Fig. 6a;Supplementary Fig. 5). Of the 16 QTL identified across the previously mapped traits, 14 were recovered by NemaScan using either the INBRED or LOCO approaches. Similar to prior results (Fig. 1c), mappings using the INBRED approach exhibited lower genomic inflation than those mappings that used the LOCO approach (Fig. 6b). We also observed that mappings that used the INBRED approach exhibited slightly lower genomic inflation than cegwas2-nf. As a result, NemaScan mappings that made use of the INBRED approach yielded the lowest concordance of overall genetic architectures for four of the five traits, including abamectin resistance for which no significant associations were detected (Fig. 6a).

Previously, two abamectin resistance QTL were mapped to the left (VL) and right (VR) arms of chromosome V. These same QTL, as well as an additional QTL on chromosome IV, were resolved using the LOCO approach in NemaScan. However, no QTL were detected using the INBRED approach, which caused the least genomic inflation for that trait (Fig. 6b). The VR QTL overlaps with the glutamate-gated chloride channel subunit *glc-1*, which underlies some macrocyclic lactone resistance in *C. elegans* (Ghosh *et al.* 2012; Burga *et al.* 2019). The VL QTL is novel and, from NIL validation experiments, has a demonstrated quantitative effect on abamectin resistance. However, this QTL resides within a hyper-divergent region, which made candidate gene identification difficult despite functional recapitulation of the QTL region for the trait of interest (Evans, Wit, *et al.* 2021). Given that both chromosome V QTL were experimentally validated, these results provide a useful example of how the LOCO approach increases the likelihood of detecting QTL within populations or specific regions of the genome subject to the effects of population stratification and subsequent proximal contamination (Listgarten *et al.* 2012). These findings confirm that NemaScan has sufficient detection power to robustly recapture experimentally validated QTL segregating in diverse *C. elegans* populations of varying size and composition.

## Discussion

### GWAS as a tool for QTL discovery in C. elegans

The *C. elegans* community has contributed steadily to the catalog of species-wide genetic variation. As the number of genetically characterized unique strains expands the CeNDR collection, we learn more about genomic patterns of diversity from all over the world. The prospects for using GWA mapping to dissect the genetic underpinnings of complex traits have improved in tandem. Although the community has successfully employed GWA mappings in *C. elegans* to discover novel genes related to a variety of traits, we lack a robust characterization of the power and precision with which this resource is equipped to detect QTL. Evaluating population-based genetic resources using simulations for other organisms has provided key benchmarks for their respective communities (Kover *et al.* 2009; Bennett *et al.* 2010; King, Macdonald, *et al.* 2012; King, Merkes, *et al.* 2012; Bouchet *et al.* 2017; Gage *et al.* 2018; Keele *et al.* 2019). The burgeoning *C. elegans* quantitative genetics community has applied GWA mapping to a growing collection of wild strains and identified genetic variants linked to complex traits with novel biomedical and evolutionary implications (Evans, van Wijk, *et al.* 2021). In the simulations presented here, we systematically tested a robust framework for GWA against a variety of genetic architectures and sample populations to contextualize past, present, and future studies using CeNDR. However, some important limitations of our simulation framework have implications in real populations.

First, simulated causal variants were selected from the minor allele frequency and LD-filtered variant set, meaning that all QTL are perfectly tagged and at >5% frequency in the population, upwardly biasing their detection in simulations. In practice, GWAS may underestimate the effects of rare QTV imperfectly tagged by filtered variants or fail to detect these variants altogether. Future work should prioritize rare variant detection, especially given their implied frequency in divergent populations (Fig. 4c). Second, effects assigned to simulated causal variants were drawn from a *Gamma* (*k = *0.4, *θ  *= 1.66) distribution (Supplementary Fig. 3) creating genetic architectures heavily biased against detection of causal alleles with very small effects. In practice, traits supported by fewer QTL of greater effect will be more amenable to GWA mapping, even at low heritability (Fig. 2c). Finally, because LD is pervasive in *C. elegans*, we define QTL in our pipeline as a region of interest defined by user-provided grouping and extension parameters (see *Materials and Methods*). The default values for these parameters in both the mapping and simulation profile are conservative in order to provide experimenters with a realistic starting point for validation experiments using classical genetic crosses. In practice, the user may specify smaller values for both parameters in order to resolve a larger number of QTL, more of which may be conditionally dependent because of LD. In spite of these limitations, we hope to provide the community with a flexible platform for QTL detection and simulation-based performance evaluation. Complementary resources for quantitative genetics in *C. elegans* also address some of these limitations. The CeMEE panel is now composed of over 1,000 lines derived from 16 founder strains. Each source population comprising the panel was reared under varying demographic and evolutionary regimes, and together, the panel offers precise QTL localization and sufficient power to identify epistatic interactions (Noble *et al.* 2017, 2021). Provided the same QTL alleles segregate in each population, we see substantial opportunities for crosstalk between GWAS using CeNDR and linkage mapping using CeMEE and mpRIL panels (Snoek *et al.* 2019) to resolve additional genetic complexity in *C. elegans*.

Similar to mapping populations of other model systems (King and Long 2017; Noble *et al.* 2017, 2021; Keele *et al.* 2019), we confirmed that the prospects of identifying QTL explaining less than 10% of overall trait variance depend primarily on three factors: (1) the number of strains being phenotyped, (2) the precision with which phenotypes can be measured, and (3) the composition of the mapping population. For instance, we observed that measuring only 100 wild strains is expected to provide almost 80% power to detect QTL that explain >40% of the phenotypic variance (Fig. 3). For many traits, it is no small feat to measure 100 strains with sufficient replication for line means to robustly represent that genetic background in a GWA mapping population. A recent GWA analysis of sperm size among 96 wild strains and N2 revealed no significant associations despite the nomination of the candidate gene *nurf-1* using segregating mutations between the N2 and LSJ1 lineages (Gimond *et al.* 2019). Another recent GWA analysis of starvation resistance using population RAD-seq read abundance in a 96 strain coculture revealed a single large-effect QTL on chromosome III whose effect was validated using NILs and was present in 11% of wild strains (Webster *et al.* 2019). These applications of GWA mappings represent mixed outcomes, providing some practical support for the conclusions of our simulations—lower sampling depths are not expected to capture entire genetic architectures, including small-effect loci or impactful alleles that segregate at low frequency (<5% of the population). Larger sample sizes (300–500 strains) and potentially fewer experimentally strenuous trait measurements are optimal to identify loci that confer more modest effects (approximately 5-10% of the phenotypic variance) with greater likelihoods (Fig. 3). Traits that can be measured in high-throughput (Hahnel *et al.* 2018; Evans, Wit, *et al.* 2021) or as intermediate traits (*e.g*., mRNA abundances) lend themselves to dissection in hundreds of strains and QTL conferring more subtle effects can be more easily resolved. At the current size of CeNDR, the primary driver of sampling depth of GWA mapping populations should be the balance between phenotyping effort for the trait of interest and the end goal of association mapping given the estimated heritability of the trait and the lower bound of the effect of QTL that will be detected (Fig. 3). In many cases, evaluating the same trait using linkage mapping in complementary populations (*i.e*., traits segregate similarly between parental strains of the cross and in the association mapping population) can validate effect sizes and provide additional support for candidates from GWA (Webster *et al.* 2019; Zdraljevic *et al.* 2019; Evans, Wit, *et al.* 2021).

### Population structure is a major determinant of performance

In this study, we also quantified the impact of mapping population structure on the power and precision of GWA mapping. We began by implementing a combination of 2 kinship matrix construction algorithms: a linear mixed model tailored toward GWAS among inbred strains (fastGWA-lmm-exact-INBRED) and a linear mixed model that corrects for genetic relatedness on all chromosomes except the chromosome containing the tested marker (fastGWA-lmm-exact-LOCO). Previous simulations of GWAS in inbred model organisms have demonstrated an increase in power by excluding markers in LD with the tested marker (Cheng *et al.* 2013). This feature is attractive for application in *C. elegans* because intra- and interchromosomal LD is pervasive in the *C. elegans* genome (Barrière and Félix 2005; Cutter 2006; Rockman and Kruglyak 2009; Cook *et al.* 2017). In comparing mappings derived from (1) choosing strains from CeNDR at random, (2) swept strains, and (3) divergent strains of equal sampling depth, we confirmed that the most power to map QTL was provided by sampling swept strains (Fig. 4a). We also found from these comparisons that the empirical FDR among the divergent strain mappings was significantly higher than the swept strain mappings when a single QTL was simulated (Fig. 5a). This result aligns with outcomes of past GWA analyses in model organisms, wherein mappings among structured populations provided less specific inference of genetic architectures (Kang *et al.* 2008). *Caenorhabditis elegans* populations also harbor highly variable patterns of genetic variation across the genome in these distinct populations, which contribute to subtle differences in local performance and inference of associations (Fig. 5) such as that mapped for abamectin (Fig. 6). However, we chose only one collection of strains to represent both divergent and swept mapping populations when considering local performance differences, which limits the general extensibility of these particular benchmarks in other populations. As different combinations of strains with varying landscapes of selective sweeps and hyper-divergent regions are tested, we will learn more about the relative influences of these regions on performance. Before concluding that an experimenter’s particular mapping population will be less powerful because it contains many divergent strains, one is advised to perform their own population-specific simulations. Below, we outline some limitations to pursuing GWA in only swept strains in certain contexts.

First, trait heritability is a major driver of detection power, which means that if the phenotype of interest does not vary significantly among swept strains, the prospects for mapping its genetic architecture heavily rely on low experimental noise. For this reason, in order to estimate the heritability of an experimenter’s trait of interest, we recommend that they measure the trait of interest and estimate its heritability in a smaller collection of *C. elegans* strains representative of the intended mapping population prior to its more extensive analysis. Divergent strains have been shown to exhibit distinct population-wide phenotypic differences from swept strains (Zhang *et al.* 2021) and therefore might be expected to contribute significantly to estimates of narrow-sense heritability of other traits. Second, swept populations will be enriched for alleles that have arisen relatively recently on swept haplotypes. Some QTL will be slightly more common in the population in swept populations (Fig. 4c), but swept populations provide a limited view of whether these QTL identified are meaningful in divergent populations that are more representative of the ancestral niche of *C. elegans* (Crombie *et al.* 2019; Lee *et al.* 2019, 2021). We know of many examples where strains more closely associated with human colonization and laboratory domestication express trait differences uncharacteristic of “wild” *C. elegans* strains (Sterken *et al.* 2015; Schulenburg and Félix 2017). Third, one kinship matrix construction algorithm used in our GWA platform was designed, in part, to collapse high relatedness among inbred individuals by creating sparse genetic covariance. This calculation is expected to provide more power in swept populations than divergent populations because the covariance among swept strains will be small enough for the algorithm to collapse more often than among divergent strains.

An instructive comparison to the improvement of *C. elegans* GWAS is the successes of identifying disease risk alleles in diverse human cohorts. *Trans*-ethnic GWAS has successfully identified common variants linked to complex human diseases by leveraging rich data and population sizes (Pendergrass *et al.* 2019; Wojcik *et al.* 2019; Hu *et al.* 2021). However, generalized predictions of disease risk in the form of polygenic risk scores suffer from sampling bias, genetic heterogeneity, and varying frequencies of risk alleles among distinct subpopulations (Li and Keating 2014; Márquez-Luna *et al.* 2017; Martin *et al.* 2019, 2020). As the community sampling of diverse *C. elegans* strains grows, GWAS will provide more power to detect QTL with more modest effects, and we will achieve more success in identifying common genetic variants linked to complex traits. However, one advantage of *C. elegans* is that complementary techniques for quantitative genetics are easily achievable and essential for validating candidate loci from GWA mappings. NILs and RILs can be derived from individual strains with large phenotypic contrasts and used for fine mapping alleles, making hypothesis-driven inferences of GWA candidate gene identification and functional tests more addressable than could be hoped for in many other species. As genomic resources for comparative evolutionary studies in *C. elegans* grow, we will characterize hyper-divergent regions more completely so that variants identified in GWA within these regions can be more confidently nominated as candidates. Furthermore, future endeavors of GWA mapping should explicitly control for the extensive population structure present among divergent strains using statistical techniques being actively applied to significantly larger cohorts of stratified human populations (Wojcik *et al.* 2019).

## Data availability

The simulation and mapping profiles of NemaScan are available at https://github.com/AndersenLab/NemaScan and are accessible with the same pipeline. In addition, we provide all parameter specifications used to generate the mappings (Supplementary Table 1). All code and data used to replicate the data analysis and figures presented are available for download at https://github.com/AndersenLab/nemascan_manuscript. All variant calls, hyper-divergent region calls, and selective sweep haplotype calls are available at https://www.elegansvariation.org/data/release/20210121. Finally, prospective users are also encouraged to use NemaScan to perform their own mappings at https://www.elegansvariation.org/mapping/perform-mapping/.

Supplemental material is available at *G3* online.

## Supplementary Material

jkac114_Supplementary_Data

jkac114_Supplementary_Figure_1

jkac114_Supplementary_Figure_2

jkac114_Supplementary_Figure_3

jkac114_Supplementary_Figure_4

jkac114_Supplementary_Figure_5

jkac114_Supplementary_Table_1

jkac114_Supplementary_Table_2

jkac114_Supplementary_Table_3

jkac114_Supplementary_Table_4

jkac114_Supplementary_Table_5

jkac114_Supplementary_Table_6

jkac114_Supplementary_Table_7

jkac114_Supplementary_Table_8

jkac114_Supplementary_Table_9

jkac114_Supplementary_Table_10
