# Supplementary material for: Evaluating the power and limitations of genome-wide association studies in Caenorhabditis elegans
Source: G3 (Bethesda). 2022 May 10;12(7):jkac114. doi: 10.1093/g3journal/jkac114 (PMC9258552; doi:10.1093/g3journal/jkac114)
Supplement: jkac114_Supplementary_Data [file jkac114_supplementary_data.pdf]

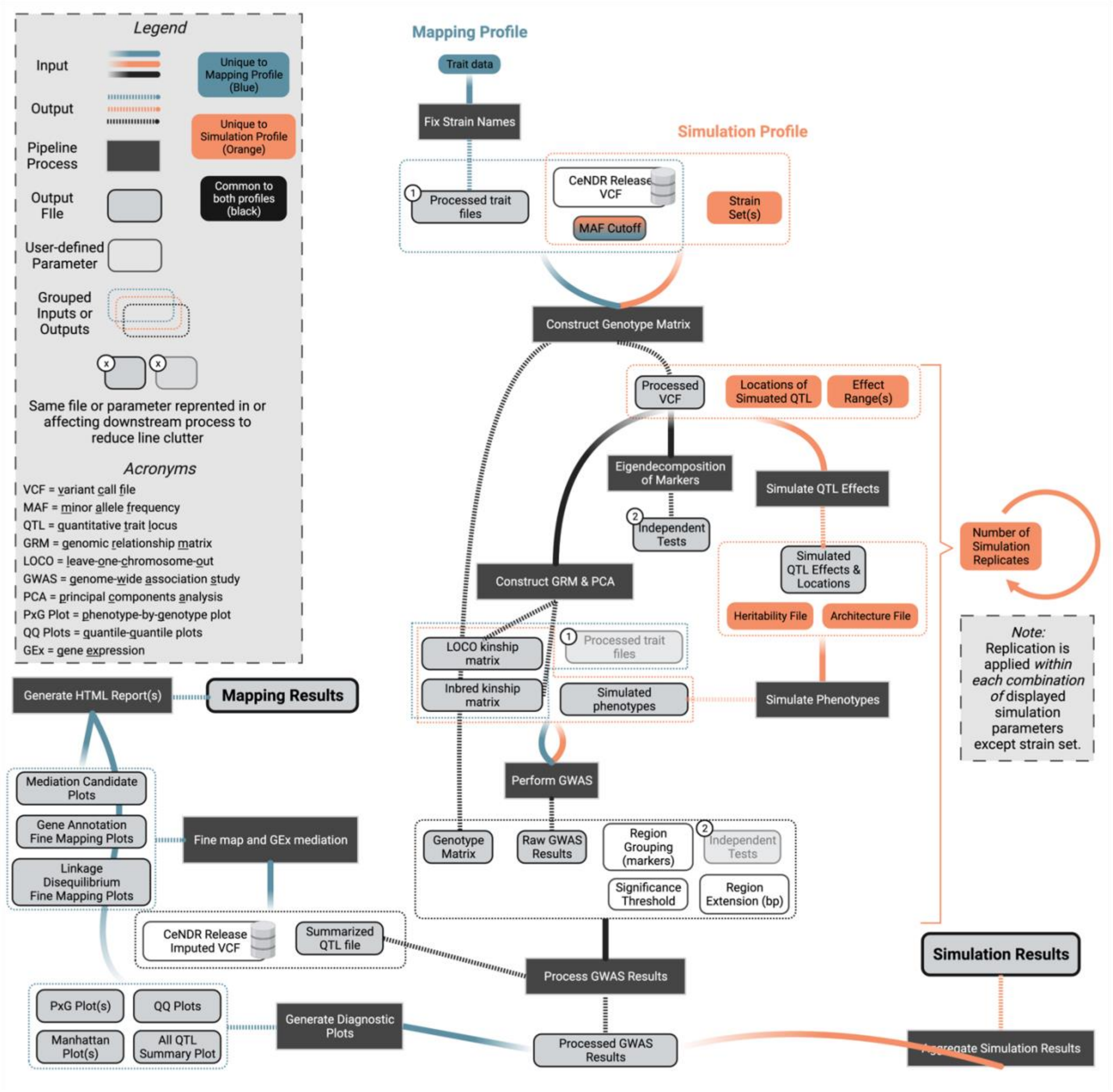


Supplemental Figure 1: Flowchart describing inputs, outputs, and processes in the mapping and simulation profiles of NemaScan.

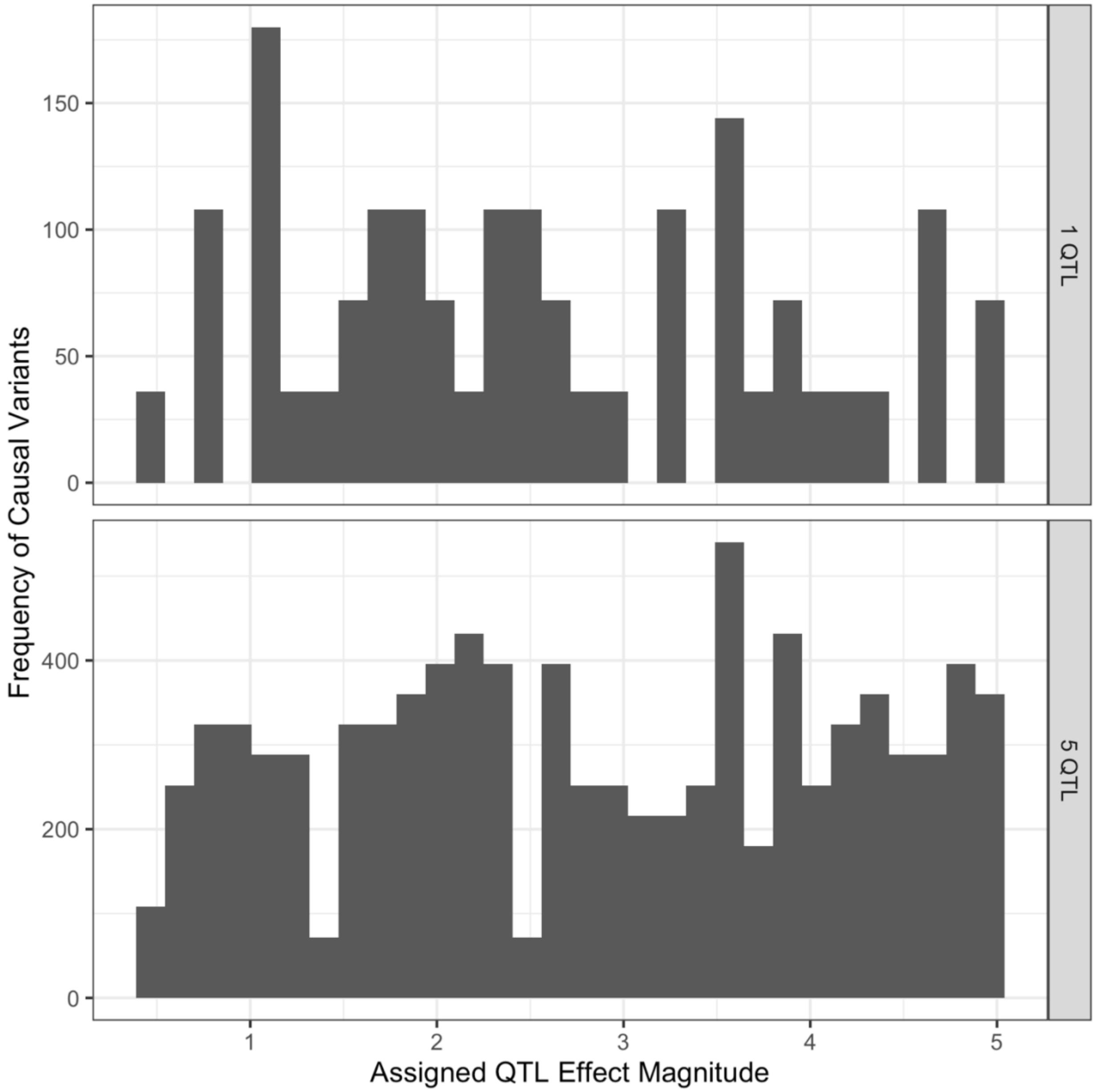


Supplemental Figure 2: Effect size distribution of simulations comparing algorithm performance

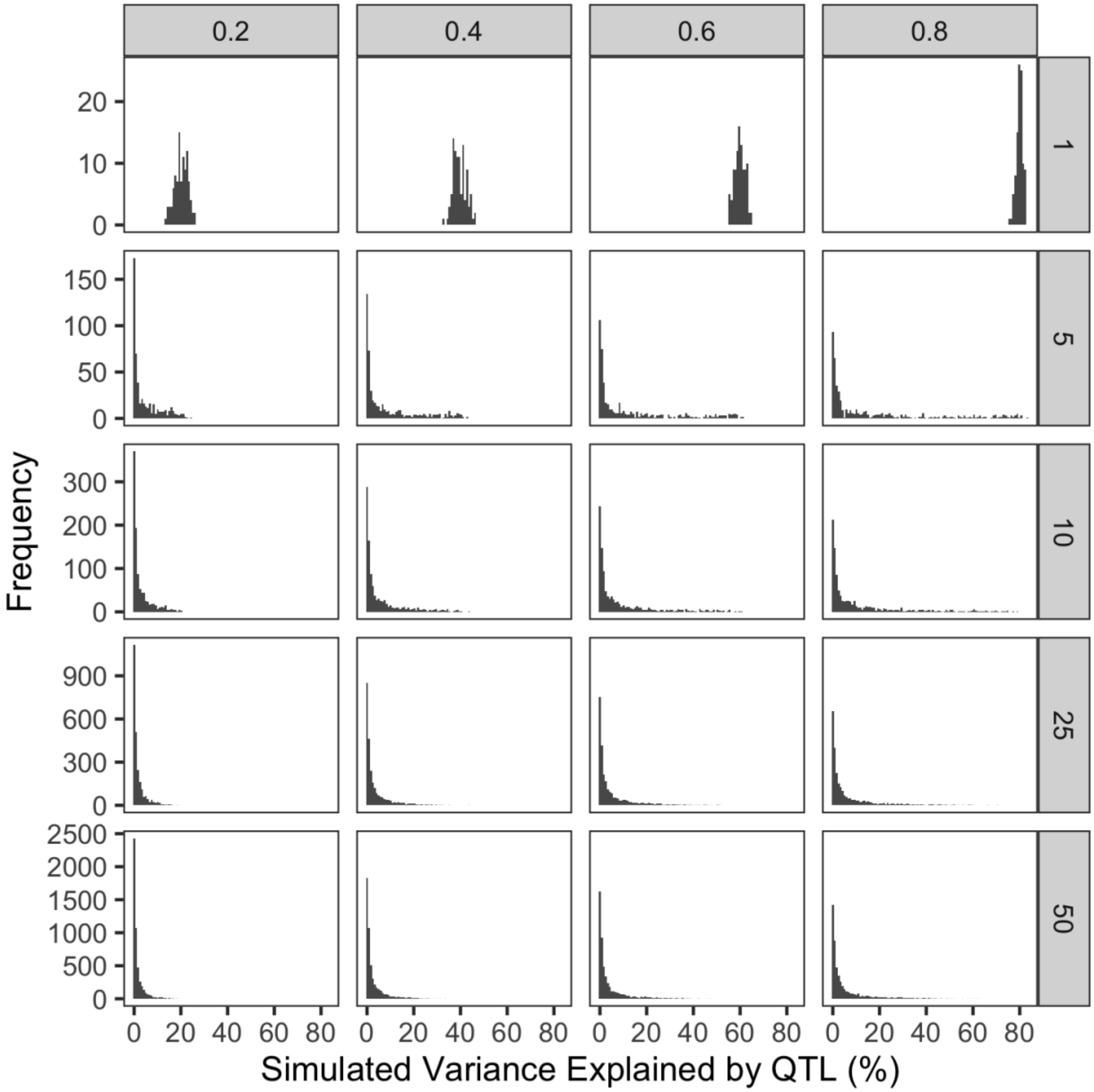


Supplemental Figure 3: Distributions of simulated QTL effects expressed as the fraction of phenotypic variance explained. Horizontal panels denote the number of simulated QTL per trait and vertical panels denote the heritability of each simulated trait.

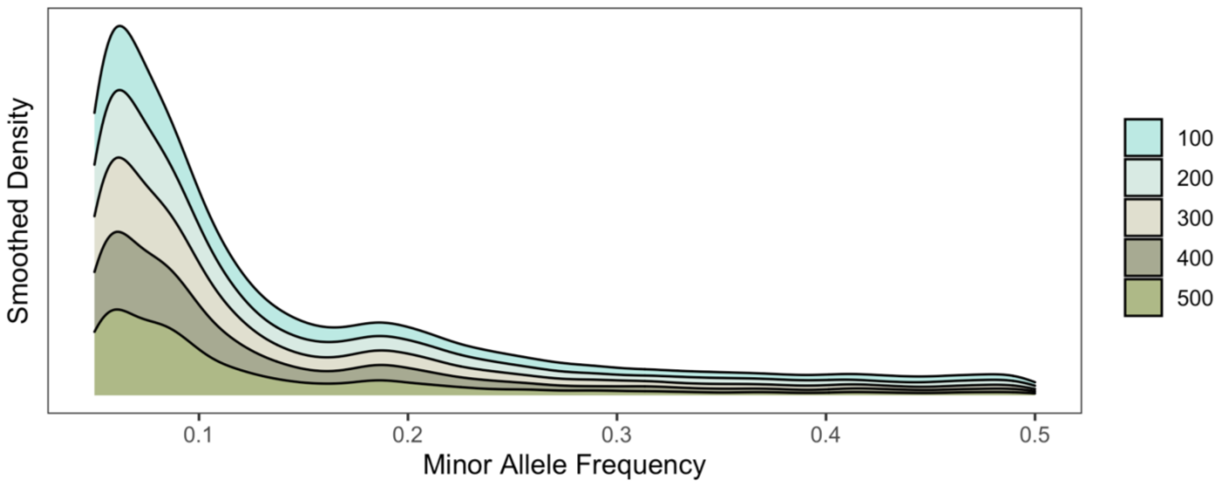


Supplemental Figure 4: Distributions of all simulated QTL minor allele frequencies among mapping populations of increasing size

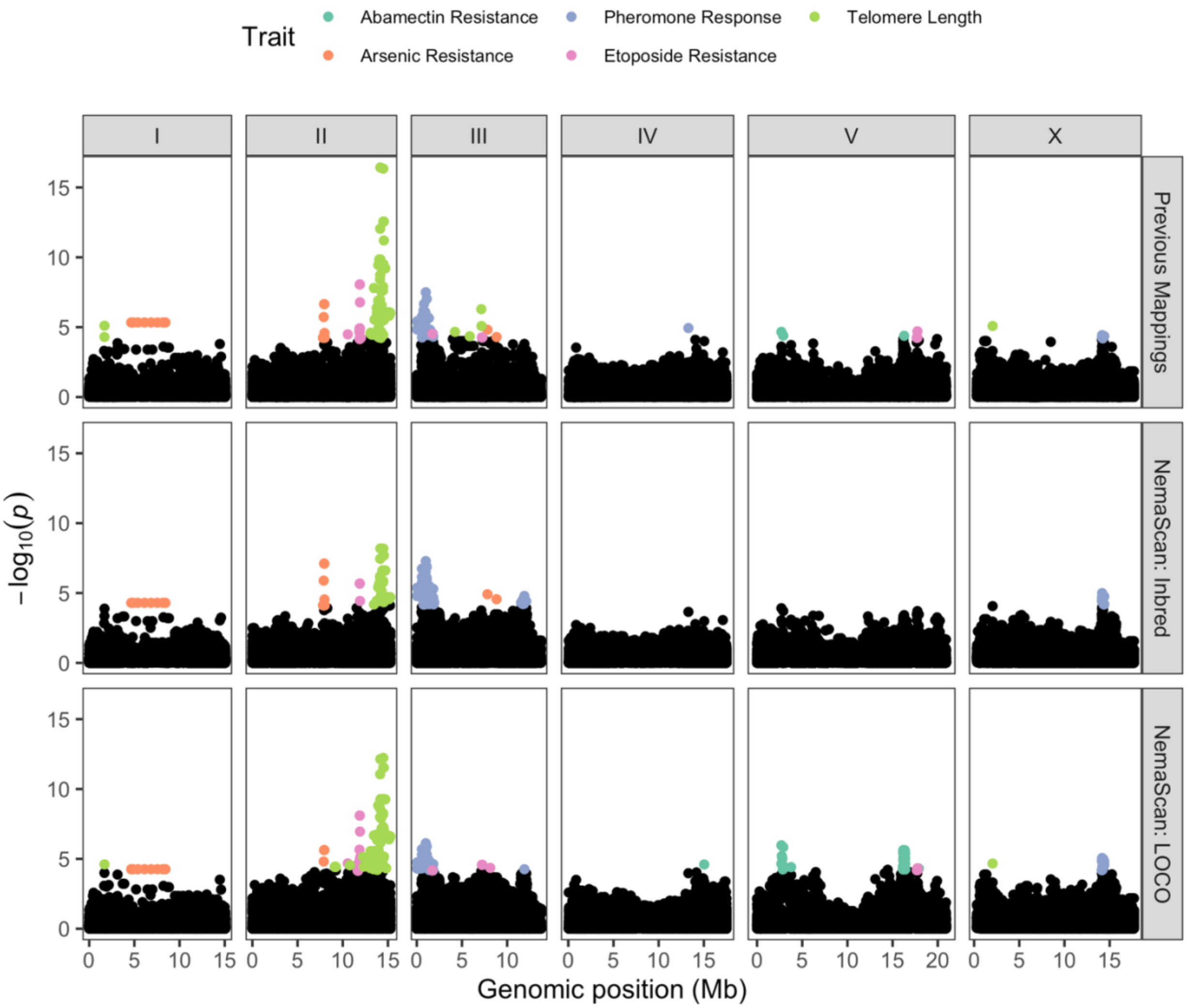


Supplemental Figure 5: Manhattan plots of previous GWA mappings and NemaScan mappings. Markers exceeding the multiple testing correction threshold are colored according to the mapped trait of interest.

| Figure | Number of Simulated QTL per Trait | Trait Heritability (h2) | Effect Distribution | Strain Set | Number of Strains | Simulated QTL Locations | Grouping Replicated | Approximate Replication per Group |
|---|---|---|---|---|---|---|---|---|
| Figure 1 | 1; 5 | 0.1; 0.2; 0.3; 0.4; 0.5; 0.6; 0.7; 0.8; 0.9 | 0.5-5 | Hahnel et. al. 2018 | 203 | Genome-wide | Heritability x Number of QTL | 50 |
| Figure 2 | 1; 5; 10; 25; 50 | 0.2; 0.4; 0.6; 0.8 | Gamma (0.4,1.66) | CeNDR Strains | 540 | Genome-wide | Heritability x Number of QTL | 100 |
| Figure 3 | 5 | 0.8 | Gamma (0.4,1.66) | Subsampled CeNDR Strains | 100; 200; 300; 400; 500 | Genome-wide | Subsampled Population | 50 |
| Figure 4 | 5 | 0.8 | Gamma (0.4,1.66) | Swept Subsampled CeNDR Strains; Divergent Subsampled CeNDR Strains; Subsampled CeNDR Strains | 144 | Genome-wide | Subsampled Population | 30 |
| Figure 5 | 1 | 0.2; 0.5; 0.8 | Uniform | All Divergent Strains; Select Swept Strains | 183; 182 | Chromosome-Specific Arms and Centers; Population-specific hyperdivergent regions | Heritability x Strain Set x Chromosome Region x Divergence x Chromosome | 100 |

Supplemental Table 1: Simulation summary

| Trait Heritability | Number of Simulated QTL | Group 1 | Group 2 | n1 | n2 | Adjusted p-value | Significance |
|---|---|---|---|---|---|---|---|
| 0.1 | 1 | EMMA | fastGWA-lmm-exact | 50 | 50 | 1 | ns |
| 0.1 | 1 | EMMA | fastGWA-lmm-exact-INBRED | 50 | 50 | 1 | ns |
| 0.1 | 1 | EMMA | fastGWA-lmm-exact-LOCO | 50 | 50 | 0.78223 | ns |
| 0.1 | 1 | fastGWA-lmm-exact | fastGWA-lmm-exact-INBRED | 50 | 50 | 1 | ns |
| 0.1 | 1 | fastGWA-lmm-exact | fastGWA-lmm-exact-LOCO | 50 | 50 | 0.41072 | ns |
| 0.1 | 1 | fastGWA-lmm-exact-INBRED | fastGWA-lmm-exact-LOCO | 50 | 50 | 0.78223 | ns |
| 0.2 | 1 | EMMA | fastGWA-lmm-exact | 50 | 50 | 1 | ns |
| 0.2 | 1 | EMMA | fastGWA-lmm-exact-INBRED | 50 | 50 | 0.44228 | ns |
| 0.2 | 1 | EMMA | fastGWA-lmm-exact-LOCO | 50 | 50 | 1 | ns |
| 0.2 | 1 | fastGWA-lmm-exact | fastGWA-lmm-exact-INBRED | 50 | 50 | 1 | ns |
| 0.2 | 1 | fastGWA-lmm-exact | fastGWA-lmm-exact-LOCO | 50 | 50 | 1 | ns |
| 0.2 | 1 | fastGWA-lmm-exact-INBRED | fastGWA-lmm-exact-LOCO | 50 | 50 | 0.76257 | ns |
| 0.3 | 1 | EMMA | fastGWA-lmm-exact | 50 | 50 | 1 | ns |
| 0.3 | 1 | EMMA | fastGWA-lmm-exact-INBRED | 50 | 50 | 0.60446 | ns |
| 0.3 | 1 | EMMA | fastGWA-lmm-exact-LOCO | 50 | 50 | 1 | ns |
| 0.3 | 1 | fastGWA-lmm-exact | fastGWA-lmm-exact-INBRED | 50 | 50 | 1 | ns |
| 0.3 | 1 | fastGWA-lmm-exact | fastGWA-lmm-exact-LOCO | 50 | 50 | 1 | ns |
| 0.3 | 1 | fastGWA-lmm-exact-INBRED | fastGWA-lmm-exact-LOCO | 50 | 50 | 0.60446 | ns |
| 0.1 | 5 | EMMA | fastGWA-lmm-exact | 50 | 50 | 1 | ns |
| 0.1 | 5 | EMMA | fastGWA-lmm-exact-INBRED | 50 | 50 | 1 | ns |
| 0.1 | 5 | EMMA | fastGWA-lmm-exact-LOCO | 50 | 50 | 1 | ns |
| 0.1 | 5 | fastGWA-lmm-exact | fastGWA-lmm-exact-INBRED | 50 | 50 | 1 | ns |
| 0.1 | 5 | fastGWA-lmm-exact | fastGWA-lmm-exact-LOCO | 50 | 50 | 1 | ns |
| 0.1 | 5 | fastGWA-lmm-exact-INBRED | fastGWA-lmm-exact-LOCO | 50 | 50 | 1 | ns |
| 0.2 | 5 | EMMA | fastGWA-lmm-exact | 50 | 50 | 1 | ns |
| 0.2 | 5 | EMMA | fastGWA-lmm-exact-INBRED | 50 | 50 | 1 | ns |
| 0.2 | 5 | EMMA | fastGWA-lmm-exact-LOCO | 50 | 50 | 1 | ns |
| 0.2 | 5 | fastGWA-lmm-exact | fastGWA-lmm-exact-INBRED | 50 | 50 | 1 | ns |
| 0.2 | 5 | fastGWA-lmm-exact | fastGWA-lmm-exact-LOCO | 50 | 50 | 1 | ns |
| 0.2 | 5 | fastGWA-lmm-exact-INBRED | fastGWA-lmm-exact-LOCO | 50 | 50 | 1 | ns |
| 0.3 | 5 | EMMA | fastGWA-lmm-exact | 50 | 50 | 1 | ns |
| 0.3 | 5 | EMMA | fastGWA-lmm-exact-INBRED | 50 | 50 | 1 | ns |
| 0.3 | 5 | EMMA | fastGWA-lmm-exact-LOCO | 50 | 50 | 1 | ns |
| 0.3 | 5 | fastGWA-lmm-exact | fastGWA-lmm-exact-INBRED | 50 | 50 | 1 | ns |
| 0.3 | 5 | fastGWA-lmm-exact | fastGWA-lmm-exact-LOCO | 50 | 50 | 0.5337 | ns |
| 0.3 | 5 | fastGWA-lmm-exact-INBRED | fastGWA-lmm-exact-LOCO | 50 | 50 | 0.5337 | ns |
| 0.4 | 5 | EMMA | fastGWA-lmm-exact | 50 | 50 | 0.23146 | ns |
| 0.4 | 5 | EMMA | fastGWA-lmm-exact-INBRED | 50 | 50 | 0.65345 | ns |
| 0.4 | 5 | EMMA | fastGWA-lmm-exact-LOCO | 50 | 50 | 0.3213 | ns |
| 0.4 | 5 | fastGWA-lmm-exact | fastGWA-lmm-exact-INBRED | 50 | 50 | 0.3213 | ns |
| 0.4 | 5 | fastGWA-lmm-exact | fastGWA-lmm-exact-LOCO | 50 | 50 | 0.00271 | ** |
| 0.4 | 5 | fastGWA-lmm-exact-INBRED | fastGWA-lmm-exact-LOCO | 50 | 50 | 0.19683 | ns |
| 0.5 | 5 | EMMA | fastGWA-lmm-exact | 50 | 50 | 0.00037 | *** |
| 0.5 | 5 | EMMA | fastGWA-lmm-exact-INBRED | 50 | 50 | 0.14917 | ns |
| 0.5 | 5 | EMMA | fastGWA-lmm-exact-LOCO | 50 | 50 | 0.14917 | ns |
| 0.5 | 5 | fastGWA-lmm-exact | fastGWA-lmm-exact-INBRED | 50 | 50 | 0.04479 | * |
| 0.5 | 5 | fastGWA-lmm-exact | fastGWA-lmm-exact-LOCO | 50 | 50 | 0 | **** |
| 0.5 | 5 | fastGWA-lmm-exact-INBRED | fastGWA-lmm-exact-LOCO | 50 | 50 | 0.00369 | ** |
| 0.6 | 5 | EMMA | fastGWA-lmm-exact | 50 | 50 | 0 | **** |
| 0.6 | 5 | EMMA | fastGWA-lmm-exact-INBRED | 50 | 50 | 0.22415 | ns |
| 0.6 | 5 | EMMA | fastGWA-lmm-exact-LOCO | 50 | 50 | 0.0589 | ns |
| 0.6 | 5 | fastGWA-lmm-exact | fastGWA-lmm-exact-INBRED | 50 | 50 | 2.00E-04 | *** |
| 0.6 | 5 | fastGWA-lmm-exact | fastGWA-lmm-exact-LOCO | 50 | 50 | 0 | **** |
| 0.6 | 5 | fastGWA-lmm-exact-INBRED | fastGWA-lmm-exact-LOCO | 50 | 50 | 0.00207 | ** |
| 0.7 | 5 | EMMA | fastGWA-lmm-exact | 50 | 50 | 0 | **** |
| 0.7 | 5 | EMMA | fastGWA-lmm-exact-INBRED | 50 | 50 | 0.48648 | ns |
| 0.7 | 5 | EMMA | fastGWA-lmm-exact-LOCO | 50 | 50 | 0.06525 | ns |
| 0.7 | 5 | fastGWA-lmm-exact | fastGWA-lmm-exact-INBRED | 50 | 50 | 6.00E-05 | **** |
| 0.7 | 5 | fastGWA-lmm-exact | fastGWA-lmm-exact-LOCO | 50 | 50 | 0 | **** |
| 0.7 | 5 | fastGWA-lmm-exact-INBRED | fastGWA-lmm-exact-LOCO | 50 | 50 | 0.01385 | * |
| 0.8 | 5 | EMMA | fastGWA-lmm-exact | 50 | 50 | 0 | **** |
| 0.8 | 5 | EMMA | fastGWA-lmm-exact-INBRED | 50 | 50 | 0.9322 | ns |
| 0.8 | 5 | EMMA | fastGWA-lmm-exact-LOCO | 50 | 50 | 0.00826 | ** |
| 0.8 | 5 | fastGWA-lmm-exact | fastGWA-lmm-exact-INBRED | 50 | 50 | 0 | **** |
| 0.8 | 5 | fastGWA-lmm-exact | fastGWA-lmm-exact-LOCO | 50 | 50 | 0 | **** |
| 0.8 | 5 | fastGWA-lmm-exact-INBRED | fastGWA-lmm-exact-LOCO | 50 | 50 | 0.00826 | ** |
| 0.9 | 5 | EMMA | fastGWA-lmm-exact | 50 | 50 | 0 | **** |
| 0.9 | 5 | EMMA | fastGWA-lmm-exact-INBRED | 50 | 50 | 0.27572 | ns |
| 0.9 | 5 | EMMA | fastGWA-lmm-exact-LOCO | 50 | 50 | 0.00501 | ** |
| 0.9 | 5 | fastGWA-lmm-exact | fastGWA-lmm-exact-INBRED | 50 | 50 | 3.00E-05 | **** |
| 0.9 | 5 | fastGWA-lmm-exact | fastGWA-lmm-exact-LOCO | 50 | 50 | 0 | **** |
| 0.9 | 5 | fastGWA-lmm-exact-INBRED | fastGWA-lmm-exact-LOCO | 50 | 50 | 0.00012 | *** |

Supplemental Table 2: Differences in power to detect QTL between mapping algorithms at increasing heritability for one and five underlying QTL

| Trait Heritab | Number of S | Group 1 | Group 2 | n1 | n2 | Adjusted p-v | Significance |
|---|---|---|---|---|---|---|---|
| 0.1 | 1 | EMMA | fastGWA-lm | 24 | 27 | 1 | ns |
| 0.1 | 1 | EMMA | fastGWA-lm | 24 | 25 | 1 | ns |
| 0.1 | 1 | EMMA | fastGWA-lm | 24 | 21 | 1 | ns |
| 0.1 | 1 | fastGWA-lm | fastGWA-lm | 27 | 25 | 1 | ns |
| 0.1 | 1 | fastGWA-lm | fastGWA-lm | 27 | 21 | 1 | ns |
| 0.1 | 1 | fastGWA-lm | fastGWA-lm | 25 | 21 | 1 | ns |
| 0.2 | 1 | EMMA | fastGWA-lm | 48 | 47 | 0.02171 | * |
| 0.2 | 1 | EMMA | fastGWA-lm | 48 | 44 | 0.69477 | ns |
| 0.2 | 1 | EMMA | fastGWA-lm | 48 | 47 | 0.69477 | ns |
| 0.2 | 1 | fastGWA-lm | fastGWA-lm | 47 | 44 | 0.07299 | ns |
| 0.2 | 1 | fastGWA-lm | fastGWA-lm | 47 | 47 | 0.00097 | *** |
| 0.2 | 1 | fastGWA-lm | fastGWA-lm | 44 | 47 | 0.5322 | ns |
| 0.3 | 1 | EMMA | fastGWA-lm | 50 | 49 | 0.6066 | ns |
| 0.3 | 1 | EMMA | fastGWA-lm | 50 | 48 | 0.12139 | ns |
| 0.3 | 1 | EMMA | fastGWA-lm | 50 | 50 | 0.00282 | ** |
| 0.3 | 1 | fastGWA-lm | fastGWA-lm | 49 | 48 | 0.6066 | ns |
| 0.3 | 1 | fastGWA-lm | fastGWA-lm | 49 | 50 | 0.12139 | ns |
| 0.3 | 1 | fastGWA-lm | fastGWA-lm | 48 | 50 | 0.6066 | ns |
| 0.4 | 1 | EMMA | fastGWA-lm | 50 | 50 | 1 | ns |
| 0.4 | 1 | EMMA | fastGWA-lm | 50 | 50 | 1 | ns |
| 0.4 | 1 | EMMA | fastGWA-lm | 50 | 50 | 1 | ns |
| 0.4 | 1 | fastGWA-lm | fastGWA-lm | 50 | 50 | 1 | ns |
| 0.4 | 1 | fastGWA-lm | fastGWA-lm | 50 | 50 | 1 | ns |
| 0.4 | 1 | fastGWA-lm | fastGWA-lm | 50 | 50 | 1 | ns |
| 0.5 | 1 | EMMA | fastGWA-lm | 50 | 50 | 0.01141 | * |
| 0.5 | 1 | EMMA | fastGWA-lm | 50 | 50 | 0.01141 | * |
| 0.5 | 1 | EMMA | fastGWA-lm | 50 | 50 | 0.01141 | * |
| 0.5 | 1 | fastGWA-lm | fastGWA-lm | 50 | 50 | 1 | ns |
| 0.5 | 1 | fastGWA-lm | fastGWA-lm | 50 | 50 | 1 | ns |
| 0.5 | 1 | fastGWA-lm | fastGWA-lm | 50 | 50 | 1 | ns |
| 0.6 | 1 | EMMA | fastGWA-lm | 50 | 50 | 0.11581 | ns |
| 0.6 | 1 | EMMA | fastGWA-lm | 50 | 50 | 0.36002 | ns |
| 0.6 | 1 | EMMA | fastGWA-lm | 50 | 50 | 1 | ns |
| 0.6 | 1 | fastGWA-lm | fastGWA-lm | 50 | 50 | 1 | ns |
| 0.6 | 1 | fastGWA-lm | fastGWA-lm | 50 | 50 | 0.1332 | ns |
| 0.6 | 1 | fastGWA-lm | fastGWA-lm | 50 | 50 | 0.36002 | ns |
| 0.7 | 1 | EMMA | fastGWA-lm | 50 | 50 | 0.04518 | * |
| 0.7 | 1 | EMMA | fastGWA-lm | 50 | 50 | 0.07324 | ns |
| 0.7 | 1 | EMMA | fastGWA-lm | 50 | 50 | 1 | ns |
| 0.7 | 1 | fastGWA-lm | fastGWA-lm | 50 | 50 | 1 | ns |
| 0.7 | 1 | fastGWA-lm | fastGWA-lm | 50 | 50 | 0.02181 | * |
| 0.7 | 1 | fastGWA-lm | fastGWA-lm | 50 | 50 | 0.04518 | * |
| 0.8 | 1 | EMMA | fastGWA-lm | 50 | 50 | 0.10115 | ns |
| 0.8 | 1 | EMMA | fastGWA-lm | 50 | 50 | 0.60209 | ns |
| 0.8 | 1 | EMMA | fastGWA-lm | 50 | 50 | 0.60209 | ns |
| 0.8 | 1 | fastGWA-lm | fastGWA-lm | 50 | 50 | 0.59338 | ns |
| 0.8 | 1 | fastGWA-lm | fastGWA-lm | 50 | 50 | 0.0077 | ** |
| 0.8 | 1 | fastGWA-lm | fastGWA-lm | 50 | 50 | 0.21354 | ns |
| 0.9 | 1 | EMMA | fastGWA-lm | 50 | 50 | 0.19981 | ns |
| 0.9 | 1 | EMMA | fastGWA-lm | 50 | 50 | 1 | ns |
| 0.9 | 1 | EMMA | fastGWA-lm | 50 | 50 | 0.26486 | ns |
| 0.9 | 1 | fastGWA-lm | fastGWA-lm | 50 | 50 | 0.19981 | ns |
| 0.9 | 1 | fastGWA-lm | fastGWA-lm | 50 | 50 | 0.00103 | ** |
| 0.9 | 1 | fastGWA-lm | fastGWA-lm | 50 | 50 | 0.26486 | ns |
| 0.1 | 5 | EMMA | fastGWA-lm | 6 | 10 | 1 | ns |
| 0.1 | 5 | EMMA | fastGWA-lm | 6 | 8 | 1 | ns |
| 0.1 | 5 | EMMA | fastGWA-lm | 6 | 2 | 1 | ns |
| 0.1 | 5 | fastGWA-lm | fastGWA-lm | 10 | 8 | 1 | ns |
| 0.1 | 5 | fastGWA-lm | fastGWA-lm | 10 | 2 | 1 | ns |
| 0.1 | 5 | fastGWA-lm | fastGWA-lm | 8 | 2 | 1 | ns |
| 0.2 | 5 | EMMA | fastGWA-lm | 22 | 31 | 1 | ns |
| 0.2 | 5 | EMMA | fastGWA-lm | 22 | 24 | 1 | ns |
| 0.2 | 5 | EMMA | fastGWA-lm | 22 | 19 | 1 | ns |
| 0.2 | 5 | fastGWA-lm | fastGWA-lm | 31 | 24 | 1 | ns |
| 0.2 | 5 | fastGWA-lm | fastGWA-lm | 31 | 19 | 0.67035 | ns |
| 0.2 | 5 | fastGWA-lm | fastGWA-lm | 24 | 19 | 1 | ns |
| 0.3 | 5 | EMMA | fastGWA-lm | 36 | 32 | 0.95233 | ns |
| 0.3 | 5 | EMMA | fastGWA-lm | 36 | 34 | 1 | ns |
| 0.3 | 5 | EMMA | fastGWA-lm | 36 | 42 | 1 | ns |
| 0.3 | 5 | fastGWA-lm | fastGWA-lm | 32 | 34 | 0.95233 | ns |
| 0.3 | 5 | fastGWA-lm | fastGWA-lm | 32 | 42 | 0.17422 | ns |
| 0.3 | 5 | fastGWA-lm | fastGWA-lm | 34 | 42 | 1 | ns |
| 0.4 | 5 | EMMA | fastGWA-lm | 45 | 34 | 1 | ns |
| 0.4 | 5 | EMMA | fastGWA-lm | 45 | 40 | 1 | ns |
| 0.4 | 5 | EMMA | fastGWA-lm | 45 | 49 | 1 | ns |
| 0.4 | 5 | fastGWA-lm | fastGWA-lm | 34 | 40 | 1 | ns |
| 0.4 | 5 | fastGWA-lm | fastGWA-lm | 34 | 49 | 1 | ns |
| 0.4 | 5 | fastGWA-lm | fastGWA-lm | 40 | 49 | 1 | ns |
| 0.5 | 5 | EMMA | fastGWA-lm | 49 | 38 | 1 | ns |
| 0.5 | 5 | EMMA | fastGWA-lm | 49 | 46 | 1 | ns |
| 0.5 | 5 | EMMA | fastGWA-lm | 49 | 50 | 1 | ns |
| 0.5 | 5 | fastGWA-lm | fastGWA-lm | 38 | 46 | 1 | ns |
| 0.5 | 5 | fastGWA-lm | fastGWA-lm | 38 | 50 | 1 | ns |
| 0.5 | 5 | fastGWA-lm | fastGWA-lm | 46 | 50 | 1 | ns |
| 0.6 | 5 | EMMA | fastGWA-lm | 49 | 40 | 0.69605 | ns |
| 0.6 | 5 | EMMA | fastGWA-lm | 49 | 50 | 0.796 | ns |
| 0.6 | 5 | EMMA | fastGWA-lm | 49 | 50 | 0.20467 | ns |
| 0.6 | 5 | fastGWA-lm | fastGWA-lm | 40 | 50 | 0.796 | ns |
| 0.6 | 5 | fastGWA-lm | fastGWA-lm | 40 | 50 | 0.0138 | * |
| 0.6 | 5 | fastGWA-lm | fastGWA-lm | 50 | 50 | 0.02481 | * |
| 0.7 | 5 | EMMA | fastGWA-lm | 50 | 44 | 1 | ns |
| 0.7 | 5 | EMMA | fastGWA-lm | 50 | 50 | 1 | ns |
| 0.7 | 5 | EMMA | fastGWA-lm | 50 | 50 | 1 | ns |
| 0.7 | 5 | fastGWA-lm | fastGWA-lm | 44 | 50 | 1 | ns |
| 0.7 | 5 | fastGWA-lm | fastGWA-lm | 44 | 50 | 0.32848 | ns |
| 0.7 | 5 | fastGWA-lm | fastGWA-lm | 50 | 50 | 1 | ns |
| 0.8 | 5 | EMMA | fastGWA-lm | 50 | 46 | 0.28505 | ns |
| 0.8 | 5 | EMMA | fastGWA-lm | 50 | 50 | 0.77132 | ns |
| 0.8 | 5 | EMMA | fastGWA-lm | 50 | 50 | 0.87436 | ns |
| 0.8 | 5 | fastGWA-lm | fastGWA-lm | 46 | 50 | 0.87436 | ns |
| 0.8 | 5 | fastGWA-lm | fastGWA-lm | 46 | 50 | 0.04972 | * |
| 0.8 | 5 | fastGWA-lm | fastGWA-lm | 50 | 50 | 0.28505 | ns |
| 0.9 | 5 | EMMA | fastGWA-lm | 50 | 48 | 0.77541 | ns |
| 0.9 | 5 | EMMA | fastGWA-lm | 50 | 50 | 0.77541 | ns |
| 0.9 | 5 | EMMA | fastGWA-lm | 50 | 50 | 0.44219 | ns |
| 0.9 | 5 | fastGWA-lm | fastGWA-lm | 48 | 50 | 0.74062 | ns |
| 0.9 | 5 | fastGWA-lm | fastGWA-lm | 48 | 50 | 0.06463 | ns |
| 0.9 | 5 | fastGWA-lm | fastGWA-lm | 50 | 50 | 0.74062 | ns |

Supplemental Table 3: Differences in empirical FDR between mapping algorithms at increasing heritability for one and five underlying QTL

| Sample Size | 0-2.5 | 2.5-5 | 5-7.5 | 7.5-10 | 10-20 | 20-30 | 30-40 | 40-50 | 50-60 | 60-70 | 70-80 | 80-90 |
|---|---|---|---|---|---|---|---|---|---|---|---|---|
| 100 | 0.01 ± 0.01 | 0.02 ± 0.04 | 0.04 ± 0.05 | 0.07 ± 0.08 | 0.22 ± 0.07 | 0.45 ± 0.12 | 0.57 ± 0.14 | 0.73 ± 0.19 | 0.81 ± 0.14 | 0.85 ± 0.13 | 0.88 ± 0.11 | 0.91 ± 0.18 |
| 200 | 0.02 ± 0.02 | 0.06 ± 0.05 | 0.14 ± 0.08 | 0.2 ± 0.12 | 0.41 ± 0.1 | 0.53 ± 0.13 | 0.67 ± 0.16 | 0.75 ± 0.14 | 0.86 ± 0.12 | 0.92 ± 0.09 | 0.95 ± 0.07 | 0.94 ± 0.18 |
| 300 | 0.03 ± 0.02 | 0.12 ± 0.06 | 0.23 ± 0.12 | 0.36 ± 0.17 | 0.48 ± 0.1 | 0.64 ± 0.12 | 0.7 ± 0.15 | 0.82 ± 0.15 | 0.88 ± 0.1 | 0.93 ± 0.08 | 0.96 ± 0.06 | 0.96 ± 0.16 |
| 400 | 0.04 ± 0.02 | 0.15 ± 0.08 | 0.28 ± 0.13 | 0.43 ± 0.17 | 0.52 ± 0.13 | 0.68 ± 0.13 | 0.75 ± 0.13 | 0.85 ± 0.13 | 0.9 ± 0.1 | 0.94 ± 0.08 | 0.99 ± 0.03 | 0.99 ± 0.04 |
| 500 | 0.05 ± 0.02 | 0.18 ± 0.08 | 0.35 ± 0.13 | 0.49 ± 0.16 | 0.59 ± 0.12 | 0.69 ± 0.12 | 0.78 ± 0.14 | 0.86 ± 0.14 | 0.91 ± 0.11 | 0.96 ± 0.07 | 0.99 ± 0.03 | 1 ± 0 |

Supplemental Table 4: Average power to detect QTL explaining increasing phenotypic variance (%, columns) among subsampled populations of increasing sampling depth

| Population Type | 0-2.5 | 2.5-5 | 5-7.5 | 7.5-10 | 10-20 | 20-30 | 30-40 | 40-50 | 50-60 | 60-70 | 70-80 | 80-90 |
|---|---|---|---|---|---|---|---|---|---|---|---|---|
| Divergent | 0.01 ± 0.01 | 0.03 ± 0.03 | 0.07 ± 0.07 | 0.12 ± 0.1 | 0.28 ± 0.1 | 0.56 ± 0.11 | 0.75 ± 0.14 | 0.87 ± 0.12 | 0.92 ± 0.12 | 0.96 ± 0.07 | 0.97 ± 0.07 | 1 ± 0 |
| Subsampled | 0.01 ± 0.02 | 0.04 ± 0.05 | 0.07 ± 0.07 | 0.15 ± 0.1 | 0.3 ± 0.1 | 0.52 ± 0.12 | 0.64 ± 0.13 | 0.75 ± 0.15 | 0.84 ± 0.13 | 0.88 ± 0.11 | 0.9 ± 0.08 | 0.93 ± 0.18 |
| Swept | 0 ± 0 | 0.01 ± 0.02 | 0.04 ± 0.07 | 0.12 ± 0.11 | 0.44 ± 0.11 | 0.85 ± 0.09 | 0.98 ± 0.04 | 1 ± 0.02 | 0.99 ± 0.02 | 1 ± 0.01 | 1 ± 0 | 1 ± 0 |

Supplemental Table 5: Average power to detect QTL explaining increasing phenotypic variance (%, columns) among 144 randomly sampled divergent strains, 144 randomly sampled swept strains, and 144 randomly sampled strains from the overall CeNDR population

| Simulated Divergence | Simulated Chromosome Region | I | II | III | IV | V | X |
|---|---|---|---|---|---|---|---|
| Hyperdivergent | Arm | 0.001170 | 0.028880 | 0.000680 | 0.037160 | 0.007740 | 0.075990 |
| Non-Hyperdivergent | Arm | 0.062080 | 0.011320 | 0.000220 | 0.005160 | 0.020250 | 0.000460 |
| Hyperdivergent | Center | 0.093920 | 0.191940 | 0.000000 | 0.002130 | 0.000730 | 0.001240 |
| Non-Hyperdivergent | Center | 0.250980 | 0.005060 | 0.000030 | 0.002930 | 0.000000 | 0.003450 |
| Hyperdivergent | Tip | 0.504990 | 0.595700 | | 0.740690 | 0.497570 | 0.000010 |
| Non-Hyperdivergent | Tip | 0.077100 | 0.614290 | 0.083260 | 0.063850 | 0.000110 | 0.016960 |

Supplemental Table 6: Differences in power to detect QTL between different chromosomes controlling for hyper-divergence and historic recombination groups (tips, arms, and centers)

| Strain Set | Group 1 | Group 2 | n1 | n2 | Adjusted p-value | Significance |
|---|---|---|---|---|---|---|
| swept.chr.sims | Hyperdivergent_Arm | Hyperdivergent_Center | 1859 | 1038 | 1 | ns |
| swept.chr.sims | Hyperdivergent_Arm | Hyperdivergent_Tip | 1859 | 700 | 1 | ns |
| swept.chr.sims | Hyperdivergent_Arm | Non-Hyperdivergent_Arm | 1859 | 1890 | 1 | ns |
| swept.chr.sims | Hyperdivergent_Arm | Non-Hyperdivergent_Center | 1859 | 1009 | 0.69252 | ns |
| swept.chr.sims | Hyperdivergent_Arm | Non-Hyperdivergent_Tip | 1859 | 695 | 1 | ns |
| swept.chr.sims | Hyperdivergent_Center | Hyperdivergent_Tip | 1038 | 700 | 1 | ns |
| swept.chr.sims | Hyperdivergent_Center | Non-Hyperdivergent_Arm | 1038 | 1890 | 1 | ns |
| swept.chr.sims | Hyperdivergent_Center | Non-Hyperdivergent_Center | 1038 | 1009 | 1 | ns |
| swept.chr.sims | Hyperdivergent_Center | Non-Hyperdivergent_Tip | 1038 | 695 | 1 | ns |
| swept.chr.sims | Hyperdivergent_Tip | Non-Hyperdivergent_Arm | 700 | 1890 | 1 | ns |
| swept.chr.sims | Hyperdivergent_Tip | Non-Hyperdivergent_Center | 700 | 1009 | 1 | ns |
| swept.chr.sims | Hyperdivergent_Tip | Non-Hyperdivergent_Tip | 700 | 695 | 1 | ns |
| swept.chr.sims | Non-Hyperdivergent_Arm | Non-Hyperdivergent_Center | 1890 | 1009 | 0.35341 | ns |
| swept.chr.sims | Non-Hyperdivergent_Arm | Non-Hyperdivergent_Tip | 1890 | 695 | 1 | ns |
| swept.chr.sims | Non-Hyperdivergent_Center | Non-Hyperdivergent_Tip | 1009 | 695 | 1 | ns |
| unswept.chr.sims | Hyperdivergent_Arm | Hyperdivergent_Center | 2030 | 1222 | 0 | **** |
| unswept.chr.sims | Hyperdivergent_Arm | Hyperdivergent_Tip | 2030 | 344 | 1 | ns |
| unswept.chr.sims | Hyperdivergent_Arm | Non-Hyperdivergent_Arm | 2030 | 2021 | 1 | ns |
| unswept.chr.sims | Hyperdivergent_Arm | Non-Hyperdivergent_Center | 2030 | 1285 | 0 | **** |
| unswept.chr.sims | Hyperdivergent_Arm | Non-Hyperdivergent_Tip | 2030 | 291 | 0.6635 | ns |
| unswept.chr.sims | Hyperdivergent_Center | Hyperdivergent_Tip | 1222 | 344 | 0.00147 | ** |
| unswept.chr.sims | Hyperdivergent_Center | Non-Hyperdivergent_Arm | 1222 | 2021 | 0 | **** |
| unswept.chr.sims | Hyperdivergent_Center | Non-Hyperdivergent_Center | 1222 | 1285 | 0.21109 | ns |
| unswept.chr.sims | Hyperdivergent_Center | Non-Hyperdivergent_Tip | 1222 | 291 | 0.6635 | ns |
| unswept.chr.sims | Hyperdivergent_Tip | Non-Hyperdivergent_Arm | 344 | 2021 | 1 | ns |
| unswept.chr.sims | Hyperdivergent_Tip | Non-Hyperdivergent_Center | 344 | 1285 | 0 | **** |
| unswept.chr.sims | Hyperdivergent_Tip | Non-Hyperdivergent_Tip | 344 | 291 | 0.62177 | ns |
| unswept.chr.sims | Non-Hyperdivergent_Arm | Non-Hyperdivergent_Center | 2021 | 1285 | 0 | **** |
| unswept.chr.sims | Non-Hyperdivergent_Arm | Non-Hyperdivergent_Tip | 2021 | 291 | 0.6635 | ns |
| unswept.chr.sims | Non-Hyperdivergent_Center | Non-Hyperdivergent_Tip | 1285 | 291 | 0.03955 | * |

Supplemental Table 7: Power to detect simulated in hyper-divergent regions or different parts of the chromosome within the mapping populations

| Trait Heritability | Strain Set | Group 1 | Group 2 | n1 | n2 | Adjusted p-value | Significance |
|---|---|---|---|---|---|---|---|
| 0.2 | swept.chr.sims | Hyperdivergent_Arm | Hyperdivergent_Center | 604 | 350 | 1 | ns |
| 0.2 | swept.chr.sims | Hyperdivergent_Arm | Hyperdivergent_Tip | 604 | 235 | 1 | ns |
| 0.2 | swept.chr.sims | Hyperdivergent_Arm | Non-Hyperdivergent_Arm | 604 | 618 | 1 | ns |
| 0.2 | swept.chr.sims | Hyperdivergent_Arm | Non-Hyperdivergent_Center | 604 | 326 | 1 | ns |
| 0.2 | swept.chr.sims | Hyperdivergent_Arm | Non-Hyperdivergent_Tip | 604 | 242 | 0.44771 | ns |
| 0.2 | swept.chr.sims | Hyperdivergent_Center | Hyperdivergent_Tip | 350 | 235 | 1 | ns |
| 0.2 | swept.chr.sims | Hyperdivergent_Center | Non-Hyperdivergent_Arm | 350 | 618 | 1 | ns |
| 0.2 | swept.chr.sims | Hyperdivergent_Center | Non-Hyperdivergent_Center | 350 | 326 | 1 | ns |
| 0.2 | swept.chr.sims | Hyperdivergent_Center | Non-Hyperdivergent_Tip | 350 | 242 | 0.48932 | ns |
| 0.2 | swept.chr.sims | Hyperdivergent_Tip | Non-Hyperdivergent_Arm | 235 | 618 | 1 | ns |
| 0.2 | swept.chr.sims | Hyperdivergent_Tip | Non-Hyperdivergent_Center | 235 | 326 | 0.31217 | ns |
| 0.2 | swept.chr.sims | Hyperdivergent_Tip | Non-Hyperdivergent_Tip | 235 | 242 | 1 | ns |
| 0.2 | swept.chr.sims | Non-Hyperdivergent_Arm | Non-Hyperdivergent_Center | 618 | 326 | 0.31217 | ns |
| 0.2 | swept.chr.sims | Non-Hyperdivergent_Arm | Non-Hyperdivergent_Tip | 618 | 242 | 1 | ns |
| 0.2 | swept.chr.sims | Non-Hyperdivergent_Center | Non-Hyperdivergent_Tip | 326 | 242 | 0.02386 | * |
| 0.2 | unswept.chr.sims | Hyperdivergent_Arm | Hyperdivergent_Center | 652 | 394 | 0.17654 | ns |
| 0.2 | unswept.chr.sims | Hyperdivergent_Arm | Hyperdivergent_Tip | 652 | 120 | 1.00E-05 | **** |
| 0.2 | unswept.chr.sims | Hyperdivergent_Arm | Non-Hyperdivergent_Arm | 652 | 630 | 0.47818 | ns |
| 0.2 | unswept.chr.sims | Hyperdivergent_Arm | Non-Hyperdivergent_Center | 652 | 416 | 0.14027 | ns |
| 0.2 | unswept.chr.sims | Hyperdivergent_Arm | Non-Hyperdivergent_Tip | 652 | 104 | 0.03318 | * |
| 0.2 | unswept.chr.sims | Hyperdivergent_Center | Hyperdivergent_Tip | 394 | 120 | 0 | **** |
| 0.2 | unswept.chr.sims | Hyperdivergent_Center | Non-Hyperdivergent_Arm | 394 | 630 | 0.01998 | * |
| 0.2 | unswept.chr.sims | Hyperdivergent_Center | Non-Hyperdivergent_Center | 394 | 416 | 0.84406 | ns |
| 0.2 | unswept.chr.sims | Hyperdivergent_Center | Non-Hyperdivergent_Tip | 394 | 104 | 0.00109 | ** |
| 0.2 | unswept.chr.sims | Hyperdivergent_Tip | Non-Hyperdivergent_Arm | 120 | 630 | 0.00028 | *** |
| 0.2 | unswept.chr.sims | Hyperdivergent_Tip | Non-Hyperdivergent_Center | 120 | 416 | 0 | **** |
| 0.2 | unswept.chr.sims | Hyperdivergent_Tip | Non-Hyperdivergent_Tip | 120 | 104 | 0.47783 | ns |
| 0.2 | unswept.chr.sims | Non-Hyperdivergent_Arm | Non-Hyperdivergent_Center | 630 | 416 | 0.00892 | ** |
| 0.2 | unswept.chr.sims | Non-Hyperdivergent_Arm | Non-Hyperdivergent_Tip | 630 | 104 | 0.14043 | ns |
| 0.2 | unswept.chr.sims | Non-Hyperdivergent_Center | Non-Hyperdivergent_Tip | 416 | 104 | 0.00065 | *** |
| 0.5 | swept.chr.sims | Hyperdivergent_Arm | Hyperdivergent_Center | 626 | 342 | 2.00E-05 | **** |
| 0.5 | swept.chr.sims | Hyperdivergent_Arm | Hyperdivergent_Tip | 626 | 231 | 1 | ns |
| 0.5 | swept.chr.sims | Hyperdivergent_Arm | Non-Hyperdivergent_Arm | 626 | 635 | 1 | ns |
| 0.5 | swept.chr.sims | Hyperdivergent_Arm | Non-Hyperdivergent_Center | 626 | 338 | 0.00027 | *** |
| 0.5 | swept.chr.sims | Hyperdivergent_Arm | Non-Hyperdivergent_Tip | 626 | 225 | 1 | ns |
| 0.5 | swept.chr.sims | Hyperdivergent_Center | Hyperdivergent_Tip | 342 | 231 | 0.00403 | ** |
| 0.5 | swept.chr.sims | Hyperdivergent_Center | Non-Hyperdivergent_Arm | 342 | 635 | 3.00E-05 | **** |
| 0.5 | swept.chr.sims | Hyperdivergent_Center | Non-Hyperdivergent_Center | 342 | 338 | 1 | ns |
| 0.5 | swept.chr.sims | Hyperdivergent_Center | Non-Hyperdivergent_Tip | 342 | 225 | 0.06213 | ns |
| 0.5 | swept.chr.sims | Hyperdivergent_Tip | Non-Hyperdivergent_Arm | 231 | 635 | 1 | ns |
| 0.5 | swept.chr.sims | Hyperdivergent_Tip | Non-Hyperdivergent_Center | 231 | 338 | 0.02052 | * |
| 0.5 | swept.chr.sims | Hyperdivergent_Tip | Non-Hyperdivergent_Tip | 231 | 225 | 1 | ns |
| 0.5 | swept.chr.sims | Non-Hyperdivergent_Arm | Non-Hyperdivergent_Center | 635 | 338 | 0.00051 | *** |
| 0.5 | swept.chr.sims | Non-Hyperdivergent_Arm | Non-Hyperdivergent_Tip | 635 | 225 | 1 | ns |
| 0.5 | swept.chr.sims | Non-Hyperdivergent_Center | Non-Hyperdivergent_Tip | 338 | 225 | 0.20773 | ns |
| 0.5 | unswept.chr.sims | Hyperdivergent_Arm | Hyperdivergent_Center | 666 | 405 | 0.01638 | * |
| 0.5 | unswept.chr.sims | Hyperdivergent_Arm | Hyperdivergent_Tip | 666 | 123 | 0 | **** |
| 0.5 | unswept.chr.sims | Hyperdivergent_Arm | Non-Hyperdivergent_Arm | 666 | 678 | 1 | ns |
| 0.5 | unswept.chr.sims | Hyperdivergent_Arm | Non-Hyperdivergent_Center | 666 | 415 | 0.0184 | * |
| 0.5 | unswept.chr.sims | Hyperdivergent_Arm | Non-Hyperdivergent_Tip | 666 | 99 | 0.02587 | * |
| 0.5 | unswept.chr.sims | Hyperdivergent_Center | Hyperdivergent_Tip | 405 | 123 | 0.02451 | * |
| 0.5 | unswept.chr.sims | Hyperdivergent_Center | Non-Hyperdivergent_Arm | 405 | 678 | 0.02451 | * |
| 0.5 | unswept.chr.sims | Hyperdivergent_Center | Non-Hyperdivergent_Center | 405 | 415 | 1 | ns |
| 0.5 | unswept.chr.sims | Hyperdivergent_Center | Non-Hyperdivergent_Tip | 405 | 99 | 1 | ns |
| 0.5 | unswept.chr.sims | Hyperdivergent_Tip | Non-Hyperdivergent_Arm | 123 | 678 | 0 | **** |
| 0.5 | unswept.chr.sims | Hyperdivergent_Tip | Non-Hyperdivergent_Center | 123 | 415 | 0.02222 | * |
| 0.5 | unswept.chr.sims | Hyperdivergent_Tip | Non-Hyperdivergent_Tip | 123 | 99 | 0.67914 | ns |
| 0.5 | unswept.chr.sims | Non-Hyperdivergent_Arm | Non-Hyperdivergent_Center | 678 | 415 | 0.02587 | * |
| 0.5 | unswept.chr.sims | Non-Hyperdivergent_Arm | Non-Hyperdivergent_Tip | 678 | 99 | 0.03264 | * |
| 0.5 | unswept.chr.sims | Non-Hyperdivergent_Center | Non-Hyperdivergent_Tip | 415 | 99 | 1 | ns |
| 0.8 | swept.chr.sims | Hyperdivergent_Arm | Hyperdivergent_Center | 623 | 344 | 1.00E-05 | **** |
| 0.8 | swept.chr.sims | Hyperdivergent_Arm | Hyperdivergent_Tip | 623 | 232 | 1 | ns |
| 0.8 | swept.chr.sims | Hyperdivergent_Arm | Non-Hyperdivergent_Arm | 623 | 633 | 1 | ns |
| 0.8 | swept.chr.sims | Hyperdivergent_Arm | Non-Hyperdivergent_Center | 623 | 338 | 0 | **** |
| 0.8 | swept.chr.sims | Hyperdivergent_Arm | Non-Hyperdivergent_Tip | 623 | 227 | 1 | ns |
| 0.8 | swept.chr.sims | Hyperdivergent_Center | Hyperdivergent_Tip | 344 | 232 | 0.00077 | *** |
| 0.8 | swept.chr.sims | Hyperdivergent_Center | Non-Hyperdivergent_Arm | 344 | 633 | 0 | **** |
| 0.8 | swept.chr.sims | Hyperdivergent_Center | Non-Hyperdivergent_Center | 344 | 338 | 1 | ns |
| 0.8 | swept.chr.sims | Hyperdivergent_Center | Non-Hyperdivergent_Tip | 344 | 227 | 0.0196 | * |
| 0.8 | swept.chr.sims | Hyperdivergent_Tip | Non-Hyperdivergent_Arm | 232 | 633 | 1 | ns |
| 0.8 | swept.chr.sims | Hyperdivergent_Tip | Non-Hyperdivergent_Center | 232 | 338 | 1.00E-04 | *** |
| 0.8 | swept.chr.sims | Hyperdivergent_Tip | Non-Hyperdivergent_Tip | 232 | 227 | 1 | ns |
| 0.8 | swept.chr.sims | Non-Hyperdivergent_Arm | Non-Hyperdivergent_Center | 633 | 338 | 0 | **** |
| 0.8 | swept.chr.sims | Non-Hyperdivergent_Arm | Non-Hyperdivergent_Tip | 633 | 227 | 0.58038 | ns |
| 0.8 | swept.chr.sims | Non-Hyperdivergent_Center | Non-Hyperdivergent_Tip | 338 | 227 | 0.004 | ** |
| 0.8 | unswept.chr.sims | Hyperdivergent_Arm | Hyperdivergent_Center | 697 | 402 | 1.00E-05 | **** |
| 0.8 | unswept.chr.sims | Hyperdivergent_Arm | Hyperdivergent_Tip | 697 | 98 | 1.00E-05 | **** |
| 0.8 | unswept.chr.sims | Hyperdivergent_Arm | Non-Hyperdivergent_Arm | 697 | 694 | 0.82137 | ns |
| 0.8 | unswept.chr.sims | Hyperdivergent_Arm | Non-Hyperdivergent_Center | 697 | 422 | 0 | **** |
| 0.8 | unswept.chr.sims | Hyperdivergent_Arm | Non-Hyperdivergent_Tip | 697 | 78 | 0.5219 | ns |
| 0.8 | unswept.chr.sims | Hyperdivergent_Center | Hyperdivergent_Tip | 402 | 98 | 0.28599 | ns |
| 0.8 | unswept.chr.sims | Hyperdivergent_Center | Non-Hyperdivergent_Arm | 402 | 694 | 0 | **** |
| 0.8 | unswept.chr.sims | Hyperdivergent_Center | Non-Hyperdivergent_Center | 402 | 422 | 0.82137 | ns |
| 0.8 | unswept.chr.sims | Hyperdivergent_Center | Non-Hyperdivergent_Tip | 402 | 78 | 0.82137 | ns |
| 0.8 | unswept.chr.sims | Hyperdivergent_Tip | Non-Hyperdivergent_Arm | 98 | 694 | 0 | **** |
| 0.8 | unswept.chr.sims | Hyperdivergent_Tip | Non-Hyperdivergent_Center | 98 | 422 | 0.5219 | ns |
| 0.8 | unswept.chr.sims | Hyperdivergent_Tip | Non-Hyperdivergent_Tip | 98 | 78 | 0.21659 | ns |
| 0.8 | unswept.chr.sims | Non-Hyperdivergent_Arm | Non-Hyperdivergent_Center | 694 | 422 | 0 | **** |
| 0.8 | unswept.chr.sims | Non-Hyperdivergent_Arm | Non-Hyperdivergent_Tip | 694 | 78 | 0.20355 | ns |
| 0.8 | unswept.chr.sims | Non-Hyperdivergent_Center | Non-Hyperdivergent_Tip | 422 | 78 | 0.82137 | ns |

Supplemental Table 8: Empirical FDR of mappings as a function of whether QTL were simulated in divergent regions and different chromosomal regions

| Strain Set | Simulated Divergence | Simulated Chromosome Region | Group 1 | Group 2 | n1 | n2 | Adjusted p-value | Significance |
|---|---|---|---|---|---|---|---|---|
| unswept.chr.sims | Hyperdivergent | Center | I | II | 105 | 100 | 0.01679 | * |
| unswept.chr.sims | Hyperdivergent | Center | II | III | 100 | 230 | 0.002 | ** |
| unswept.chr.sims | Hyperdivergent | Center | III | V | 230 | 387 | 0.0051 | ** |
| unswept.chr.sims | Hyperdivergent | Center | III | X | 230 | 220 | 0.00646 | ** |
| unswept.chr.sims | Hyperdivergent | Tip | IV | X | 89 | 43 | 0.00446 | ** |
| unswept.chr.sims | Hyperdivergent | Tip | V | X | 156 | 43 | 0.00749 | ** |
| swept.chr.sims | Non-Hyperdivergent | Arm | II | V | 488 | 233 | 0.04509 | * |

Supplemental Table 9: Power to detect simulated QTL on different chromosomes, within hyper-divergent regions, historic recombination groups, and strain sets

| Strain Set | Simulated Divergence | Simulated Chromosome Region | Group 1 | Group 2 | n1 | n2 | Adjusted p-value | Significance |
|---|---|---|---|---|---|---|---|---|
| swept.chr.sims | Hyperdivergent | Arm | I | II | 168 | 483 | 0.00007 | **** |
| swept.chr.sims | Hyperdivergent | Arm | I | III | 168 | 359 | 0.00001 | **** |
| swept.chr.sims | Hyperdivergent | Arm | I | IV | 168 | 360 | 0.00006 | **** |
| swept.chr.sims | Hyperdivergent | Arm | I | V | 168 | 236 | 0.00002 | **** |
| swept.chr.sims | Hyperdivergent | Arm | I | X | 168 | 247 | 0 | **** |
| swept.chr.sims | Hyperdivergent | Arm | II | X | 483 | 247 | 0.00054 | *** |
| swept.chr.sims | Hyperdivergent | Arm | III | X | 359 | 247 | 0.01327 | * |
| swept.chr.sims | Hyperdivergent | Arm | IV | X | 360 | 247 | 0.00343 | ** |
| unswept.chr.sims | Hyperdivergent | Arm | III | X | 343 | 269 | 0.03806 | * |
| unswept.chr.sims | Hyperdivergent | Center | I | II | 102 | 99 | 0.00015 | *** |
| unswept.chr.sims | Hyperdivergent | Center | I | III | 102 | 221 | 0 | **** |
| unswept.chr.sims | Hyperdivergent | Center | I | V | 102 | 383 | 0 | **** |
| unswept.chr.sims | Hyperdivergent | Center | I | X | 102 | 216 | 0.00015 | *** |
| unswept.chr.sims | Hyperdivergent | Center | III | IV | 221 | 180 | 0.0002 | *** |
| unswept.chr.sims | Hyperdivergent | Center | IV | V | 180 | 383 | 0.00085 | *** |
| swept.chr.sims | Hyperdivergent | Tip | III | IV | 24 | 183 | 0.00191 | ** |
| swept.chr.sims | Hyperdivergent | Tip | III | V | 24 | 303 | 0.00099 | *** |
| swept.chr.sims | Hyperdivergent | Tip | III | X | 24 | 145 | 0.00002 | **** |
| unswept.chr.sims | Hyperdivergent | Tip | I | V | 27 | 156 | 0.00523 | ** |
| unswept.chr.sims | Hyperdivergent | Tip | III | V | 14 | 156 | 0.01308 | * |
| swept.chr.sims | Non-Hyperdivergent | Arm | I | II | 190 | 488 | 0.02112 | * |
| swept.chr.sims | Non-Hyperdivergent | Arm | I | III | 190 | 374 | 0.00002 | **** |
| swept.chr.sims | Non-Hyperdivergent | Arm | I | IV | 190 | 376 | 0.00001 | **** |
| swept.chr.sims | Non-Hyperdivergent | Arm | I | V | 190 | 232 | 0.00731 | ** |
| swept.chr.sims | Non-Hyperdivergent | Arm | I | X | 190 | 226 | 0 | **** |
| swept.chr.sims | Non-Hyperdivergent | Arm | II | X | 488 | 226 | 0.00004 | **** |
| swept.chr.sims | Non-Hyperdivergent | Arm | V | X | 232 | 226 | 0.01199 | * |
| unswept.chr.sims | Non-Hyperdivergent | Arm | I | V | 408 | 404 | 0.04823 | * |
| unswept.chr.sims | Non-Hyperdivergent | Arm | IV | V | 289 | 404 | 0.04823 | * |
| swept.chr.sims | Non-Hyperdivergent | Center | I | IV | 15 | 114 | 0.04399 | * |
| swept.chr.sims | Non-Hyperdivergent | Center | I | X | 15 | 321 | 0.01193 | * |
| swept.chr.sims | Non-Hyperdivergent | Center | II | X | 216 | 321 | 0.00542 | ** |
| swept.chr.sims | Non-Hyperdivergent | Center | III | X | 155 | 321 | 0.00028 | *** |
| unswept.chr.sims | Non-Hyperdivergent | Center | I | III | 157 | 237 | 0.00608 | ** |
| unswept.chr.sims | Non-Hyperdivergent | Center | I | V | 157 | 364 | 0.00018 | *** |
| unswept.chr.sims | Non-Hyperdivergent | Center | III | X | 237 | 185 | 0.00703 | ** |
| unswept.chr.sims | Non-Hyperdivergent | Center | IV | V | 199 | 364 | 0.02493 | * |
| unswept.chr.sims | Non-Hyperdivergent | Center | V | X | 364 | 185 | 0.00018 | *** |
| swept.chr.sims | Non-Hyperdivergent | Tip | I | III | 15 | 24 | 0.02849 | * |
| swept.chr.sims | Non-Hyperdivergent | Tip | II | III | 63 | 24 | 0.00959 | ** |
| swept.chr.sims | Non-Hyperdivergent | Tip | III | IV | 24 | 176 | 0.02297 | * |
| swept.chr.sims | Non-Hyperdivergent | Tip | III | X | 24 | 99 | 0.02148 | * |

Supplemental Table 10: Empirical FDR of mappings as a function of whether QTL were simulated on different chromosomes, within hyper-divergent regions, historic recombination groups, and strain sets
