# Supplementary figures and images for: Evaluating the power and limitations of genome-wide association studies in Caenorhabditis elegans
Source: G3 (Bethesda). 2022 May 10;12(7):jkac114. doi: 10.1093/g3journal/jkac114 (PMC9258552; doi:10.1093/g3journal/jkac114)

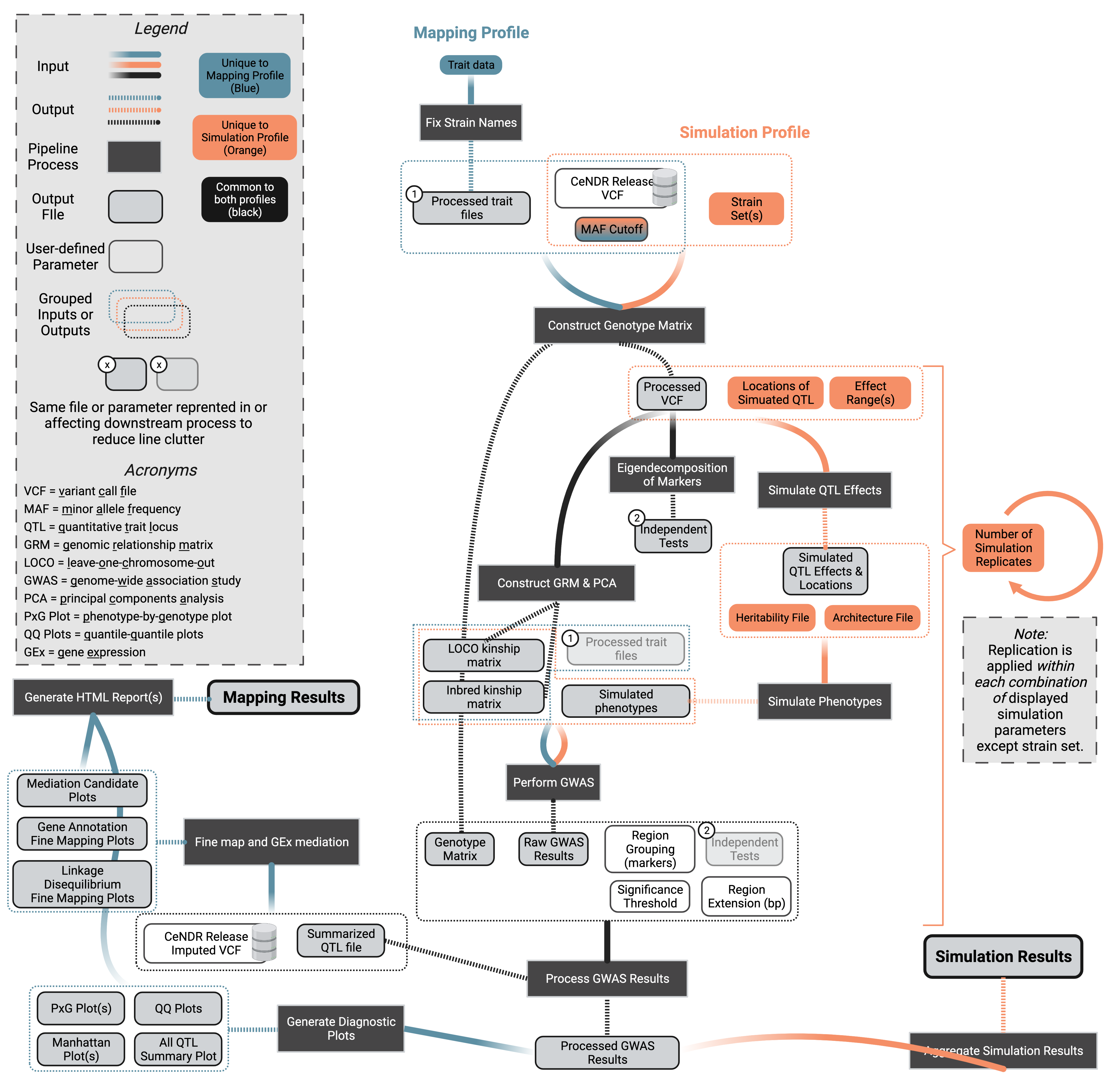

Supplement: jkac114_Supplementary_Figure_1 [file jkac114_supplementary_figure_1.zip › jkac114_Supplementary_Figure_1.png]

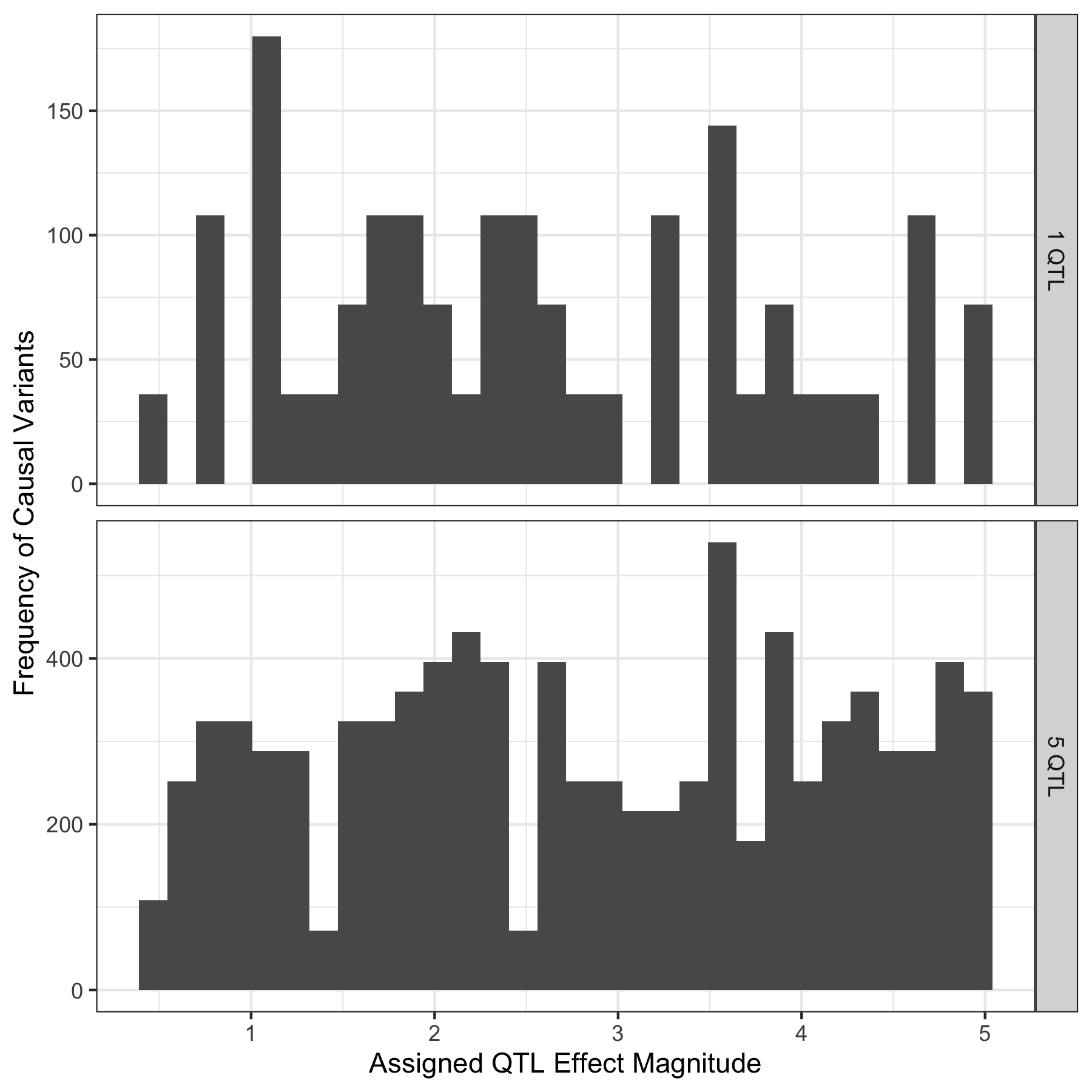

Supplement: jkac114_Supplementary_Figure_2 [file jkac114_supplementary_figure_2.zip › jkac114_Supplementary_Figure_2.png]

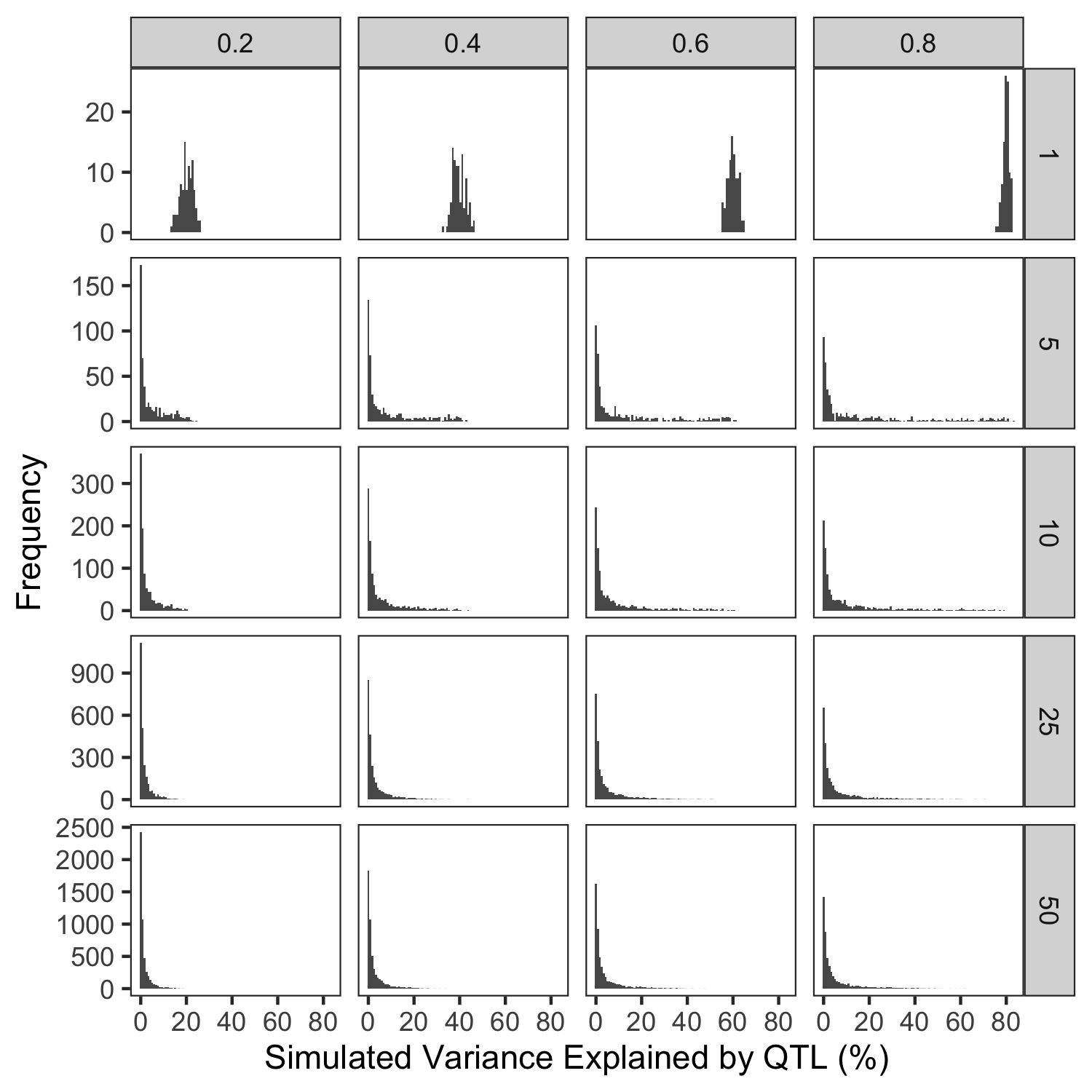

Supplement: jkac114_Supplementary_Figure_3 [file jkac114_supplementary_figure_3.zip › jkac114_Supplementary_Figure_3.png]

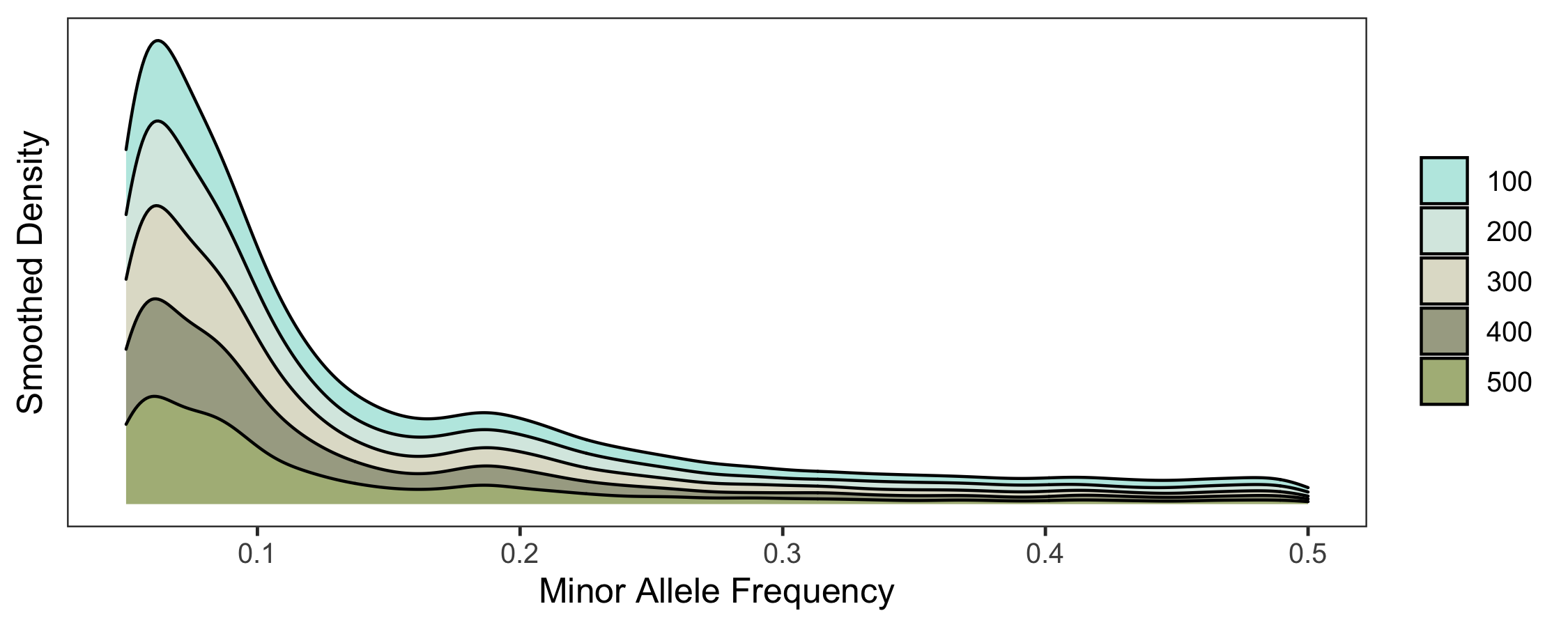

Supplement: jkac114_Supplementary_Figure_4 [file jkac114_supplementary_figure_4.zip › jkac114_Supplementary_Figure_4.png]

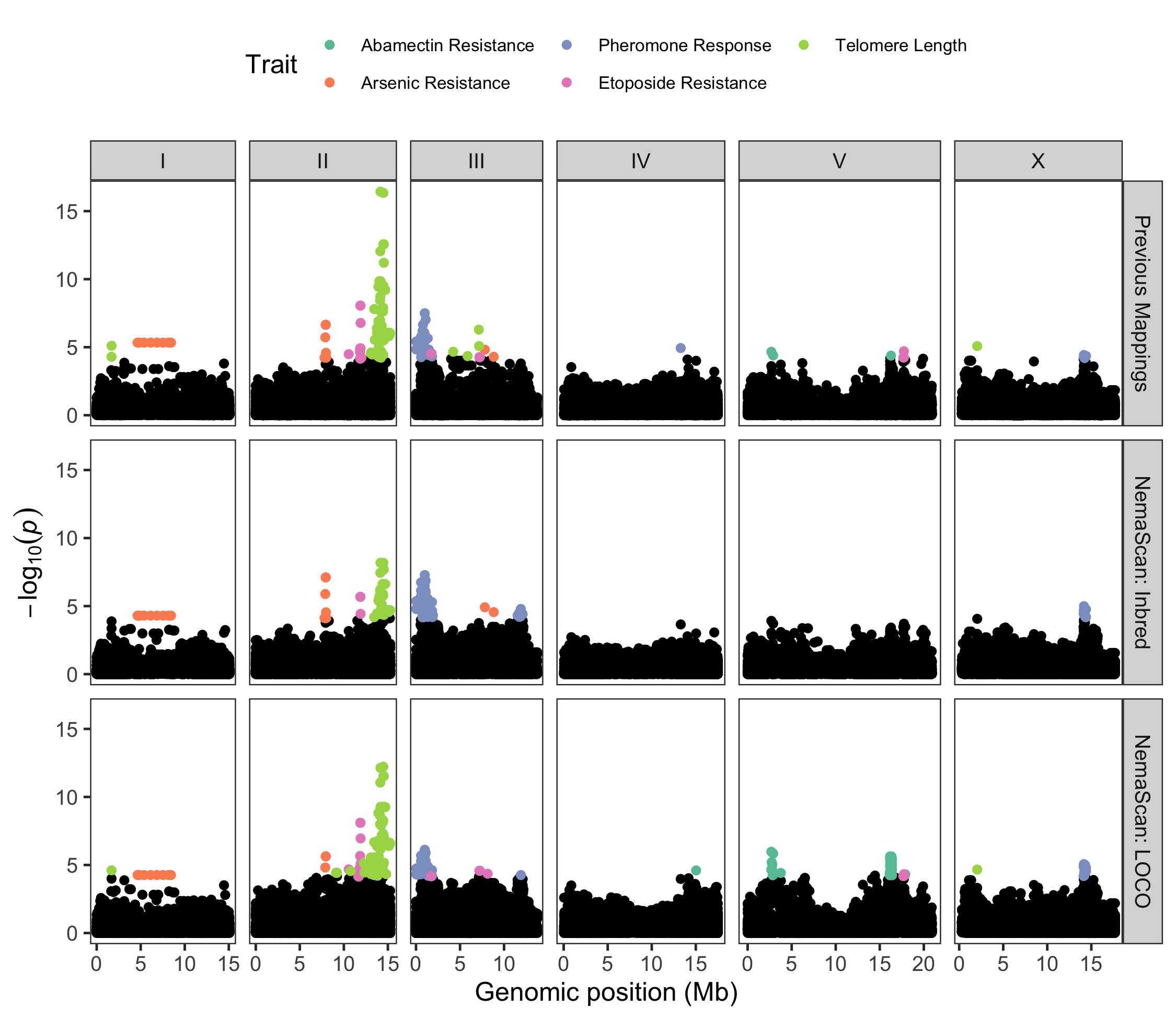

Supplement: jkac114_Supplementary_Figure_5 [file jkac114_supplementary_figure_5.zip › jkac114_Supplementary_Figure_5.png]
